# Solvothermal Template‐Induced Hierarchical Porosity in Covalent Organic Frameworks: A Pathway to Enhanced Diffusivity

**DOI:** 10.1002/adma.202415882

**Published:** 2025-01-21

**Authors:** Fabian Heck, Lars Grunenberg, Nadine Schnabel, Amelie Heilmaier, Thomas Sottmann, Liang Yao, Bettina V. Lotsch

**Affiliations:** ^1^ Max Planck Institute for Solid State Research Heisenbergstr. 1 70569 Stuttgart Germany; ^2^ Department of Chemistry Ludwig‐Maximilians‐Universität (LMU) Butenandtstr. 5–13 81377 Munich Germany; ^3^ Collaborative Research Center 1333 Universität Stuttgart Pfaffenwaldring 55 70569 Stuttgart Germany; ^4^ Institute of Physical Chemistry Pfaffenwaldring 55 70569 Stuttgart Germany; ^5^ State Key Laboratory of Luminescent Materials and Devices Institute of Polymer Optoelectronic Materials and Devices Guangdong Basic Research Centre of Excellence for Energy and Information Polymer Materials South China University of Technology Guangzhou 510640 P. R. China

**Keywords:** covalent organic frameworks, diffusion, electrochemical CO_2_ reduction, gas adsorption, hierarchical porosity, pulsed field gradient NMR, synthetic methodology

## Abstract

The rapid advancement of covalent organic frameworks (COFs) in recent years has firmly established them as a new class of molecularly precise and highly tuneable porous materials. However, compared to other porous materials, such as zeolites and metal‐organic frameworks, the successful integration of hierarchical porosity into COFs remains largely unexplored. The challenge lies in identifying appropriate synthetic methods to introduce secondary pores without compromising the intrinsic structural porosity of COFs. In this study, a template‐induced synthetic methodology is realized to facilitate the construction of hierarchically porous COFs (hCOFs). This novel approach utilizes commercially available zinc oxide nanoparticles as a hard template, enabling to increase the total pore volume of a series of *β*‐ketoenamine‐linked COFs as well as an imine‐based COF while preserving their surface areas. In addition to transmission electron microscopy and gas adsorption analyses, small‐angle X‐ray scattering and pulsed field gradient nuclear magnetic resonance techniques are employed to investigate the hierarchical porosity and diffusivity of guest molecules within hCOFs. This study demonstrates that the hierarchically porous nature of hCOFs significantly reduces diffusion limitations, thus leading to simultaneous enhancements in adsorption capacity, diffusivity, and catalytic performance.

## Graphical abstract

Hierarchically porous covalent organic frameworks (hCOFs) are constructed using a solvothermal template‐induced synthetic strategy. Commercially available zinc oxide nanoparticles are employed as hard template enabling an increase in total pore volume in a series of COFs. The hierarchically porous nature of hCOFs significantly reduces diffusion limitations, thus leading to simultaneous enhancements in adsorption capacity, diffusivity, and catalytic performance.

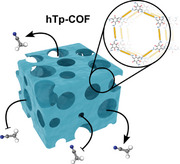

## 1 Introduction

The pursuit of hierarchically porous materials has garnered considerable attention from academic to industrial applications, due to their widespread usage in energy conversion,^[^ ^1^ ^]^ catalysis,^[^ ^2^ ^]^ adsorption^[^ ^3^ ^]^ as well as separation.^[^ ^4^ ^]^ In comparison to non‐hierarchically porous systems, the rational design of hierarchical porosity usually facilitates significant enhancement in mass transport and pore accessibility, leading to superior performance and efficiency in applications.^[^ ^5^ ^]^ Consequently, there is a strong push toward the rational development of systems with bi‐ or multimodal pore size distributions in a large number of functional materials ranging from zeolites and metal oxides to polymers and metal organic frameworks (MOFs). Among other synthetic methodologies, templating protocols and self‐assembly of pre‐formed crystallites to 3D morphologies currently prevail as prominent strategies to achieve hierarchical structuring in these materials.^[^ ^6^ ^]^ Over the last two decades, covalent organic frameworks (COFs) have undergone extensive development as a nascent class of porous materials due to the high modularity of building blocks, precisely tailored pore structures and a wide range of applications.^[^ ^7^ ^]^ Recently, there has been growing interest in designing hierarchical porosity in COFs. A well‐known reported strategy for introducing bimodal porosity at the molecular level is to utilize special topologies, such as Kagome‐type structures.^[^ ^8^ ^]^ While several COFs have been successfully demonstrated with dual porous architectures through this approach, their scope remains confined to the micro‐ and small mesopore domain, i.e. on the level of structural rather than textural pores.^[^ ^9^ ^]^ Templating methods have been widely applied to aforementioned functional materials and represent potentially promising approaches to introduce textural porosity in COFs on the larger meso‐ and macroporous scale. However, the challenge lies in finding an appropriate combination of sacrificial template and synthesis conditions that ensures both the preservation of the template's structure during COF formation and facile removal of the template without compromising the framework's integrity.^[^ ^10^ ^]^ Thomas and co‐workers achieved macro/microporous COFs through a solid‐state approach and colloidal polystyrene (PS) nanoparticles as hard template, which provide secondary macropores up to several hundreds of nanometers.^[^ ^11^ ^]^ Nonetheless, PS nanoparticles are soluble in many organic solvents, e.g. chlorobenzene or dioxane, and hence are not suitable for common solvothermal COF syntheses.^[^ ^12^ ^]^ In a recent work, silicon dioxide nanoparticles were employed as hard template in a mechanochemical approach to prepare hierarchically porous COFs (hCOFs).^[^ ^13^ ^]^ However, the general utilization of silica as sacrificial template is restricted by harsh template‐etching conditions in fluoride‐containing (NH_4_F, HF) or strong alkaline solutions, which, apart from their hazardous nature, often cause decomposition of frameworks by hydrolysis of the COF linkages. While ionic liquids have been tested as promising soft templates for hierarchical (micro/meso‐) porosity in COFs, gas adsorption studies indicated only a modest incorporation of secondary pores by this strategy.^[^ ^14^ ^]^ Therefore, a lack of innovative synthetic methodologies to propel the advancement of hCOFs persists. Furthermore, thus far, only nitrogen gas adsorption and standard electron microscopy techniques were utilized to investigate the hierarchy in porosity in the aforementioned studies. Advanced characterization methods toward elucidating the multi‐porous architecture as well as its advantages are still lacking in the field of hCOFs and require further development.

In this work, we report a facile synthetic protocol to introduce hierarchical meso/mesoporosity in a series of COFs to improve gas adsorption capacity and demonstrate orders of magnitude higher diffusivity as well as superior catalytic activity of the hierarchically porous hCOFs. Our method makes use of commercially available zinc oxide nanoparticles (ZnO NPs) as template in a fast solvothermal and catalyst‐free synthesis to create large mesopores in the material backbone. To demonstrate the generality of this template‐induced approach, the concept was applied to a series of isoreticular *β*‐ketoenamine‐linked COFs with increasing pore diameter, namely TpBz (2.0 nm), TpTPD (2.5 nm), and TpQPD (3.0 nm) and further transferred to an imine linked COF, i.e. TAPB‐MeOTP.

## 2 Results

### 2.1 Synthetic Method and Analysis

**Figure 1:**
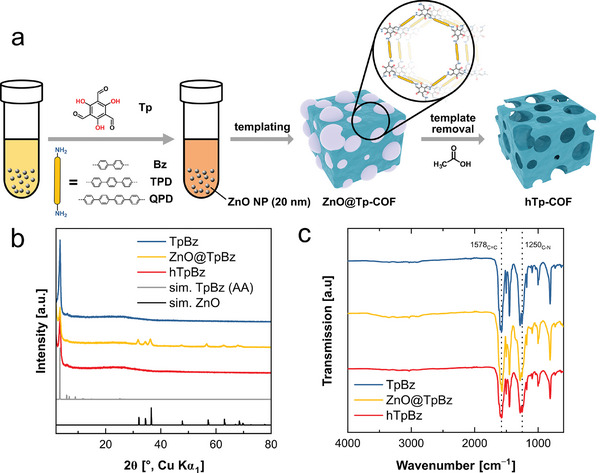
a) Synthesis scheme of template‐induced approach toward hierarchical porosity in *β*‐ketoenamine COFs. b) XRPD patterns and c) FTIR‐ATR spectra of the pristine and the hierarchical COF vs. the zinc oxide loaded version.

Zinc oxide is a well‐known n‐type inorganic semiconducting material, and has extensively been applied in photovoltaic devices, light emitting diodes, and photocatalysts.^[^ ^15^ ^]^ It shows good stability in basic media, while ZnO can be dissolved in acidic conditions. This property offers the possibility to employ ZnO as a hard template for the synthesis of hCOFs. So far, however, the combination of ZnO with COF synthesis remains largely unexplored. An exception is the work by Banerjee and co‐workers, who reported hollow tubular porous COFs using ZnO nanorod templates, although the ZnO incorporated COFs, as well as the resulting tubular porous COFs, exhibit a discernibly diminished crystallinity, as evidenced by X‐ray powder diffraction (XRPD) analysis.^[^ ^16^ ^]^ Furthermore, in comparison to the control COF, no total pore volume increase can be observed for the tubular porous frameworks. In this study, we utilized commercially available ZnO NPs (20 nm) for the preparation of hierarchically porous frameworks, and developed synthetic routes to access hCOFs without compromising the crystallinity, while simultaneously achieving a significant increase in total pore volume. In an improved synthesis method, 2,4,6‐trihydroxybenzene‐1,3,5‐tricarbaldehyde (Tp) was dissolved in DMF and slowly added to the amine linker solution via a syringe pump to form the pristine hexagonal *β*‐ketoenamine COF system in the absence of an acidic catalyst. The slow addition of Tp enhances COF quality by allowing to control COF nucleation and growth.^[^ ^17^ ^]^ For the hCOFs, Tp was added slowly to a mixture of the amine linker solution combined with a ZnO NPs suspension (2.5 wt.% in propylene glycol and isopropanol), enabling the incorporation of the ZnO template into the framework. In a second, post‐synthetic step, the obtained materials were suspended in acetic acid (6 m) to remove the ZnO NPs without harming the COF's integrity, and to expose the hierarchically porous structure (**Figure** **1**a). To verify the colloidal stability of the purchased ZnO NP suspension under synthetic conditions, a ZnO NP suspension treated under reaction conditions was compared to the pristine ZnO NPs via DLS measurements and shows insignificant changes in particle size (≈20 nm, Figure S47, Supporting Information). Next, efforts were made toward optimization of the template loading amount of the ZnO@TpBz COF, and an optimum loading, based on the relative intensities in the XRPD as a proxy for crystallinity, of 10.5 µL ZnO NP suspension per micromole of Tp was identified (Figure S4, see Supporting Information for more information). A comparison of the experimental XRPD data of TpBz, ZnO@TpBz and hTpBz is displayed in Figure 1b. The results show a narrow (100) reflection at 2*θ* = 3.5° for TpBz and minor broad reflections at 5.9° (110) and 6.8° (200), which is in line with reported patterns of TpBz‐COF obtained by acid‐catalyzed synthesis. Intensities and reflection positions coincide with the simulated pattern for an idealized eclipsed AA stacked structural model.^[^ ^18^ ^]^ The absence of stacking reflections (001) is commonly observed for this TpBz and attributed to conformational variability of the COF and varying interlayer distances, which can lead to reduced coherence between layers.^[^ ^19^ ^]^ With ZnO NPs incorporated, peaks at 2*θ* = 31.8°, 34.4°, 36.2°, 47.6°, 56.8°, 62.7°, 68.0°, 68.2° are visible in the XRPD, in addition to characteristic reflections of TpBz. These are in agreement with the simulated diffraction pattern for Wurtzite‐type ZnO.^[^ ^20^ ^]^ Upon template‐removal by an acetic acid treatment, the ZnO reflections disappeared whereas those of the COF remained, hinting at a successful etching of the template without harming the COFs integrity (Figure 1b). This was further supported by inductively coupled plasma optical emission spectroscopy (ICP‐OES) analysis, showing a reduced zinc content from 29.8 wt.% in ZnO@TpBz to 0.09 wt.% in hTpBz, demonstrating an essentially complete removal of zinc from the material upon etching (Table S1, Supporting Information). Fourier transform infrared (FT‐IR) spectra of TpBz and ZnO@TpBz show characteristic vibrations at 1578 cm^−1^ (V_C═C_) and 1250 cm^−1^ (V_C‐N_) which indicates successful formation of the *β*‐ketoenamine‐linked frameworks in the absence of acid as catalyst during the synthesis (Figure 1c).^[^ ^18^, ^21^ ^]^ These vibrations remain after acetic acid treatment in hTpBz and suggest that no chemical decomposition by the etching protocol has occurred. Similar results in XRPD, ICP‐OES and FT‐IR analyses were achieved for two additional *β*‐ketoenamine candidates with increased pore sizes, namely TpTPD^[^ ^11a^ ^]^ (Figure S1, Supporting Information) and TpQPD (Figure S2, Supporting Information). The newly developed synthetic strategy was further extended to an imine based system with TAPB‐MeOTP.^[^ ^7c^ ^]^ After the colloidal stability of the ZnO NPs in Sc(OTf)_3_ and acetonitrile was confirmed via DLS (Figure S47, Supporting Information), the hard template was combined with the reaction mixture to fabricate ZnO@TAPB‐MeOTP. Subsequent to the acetic acid etching step, similar results in XRPD analysis were obtained in accordance with the *β*‐ketoenamine series (Figure S11, Supporting Information), which illustrates the compatibility of this solvothermal based templating and etching method with other COF systems.

**Figure 2:**
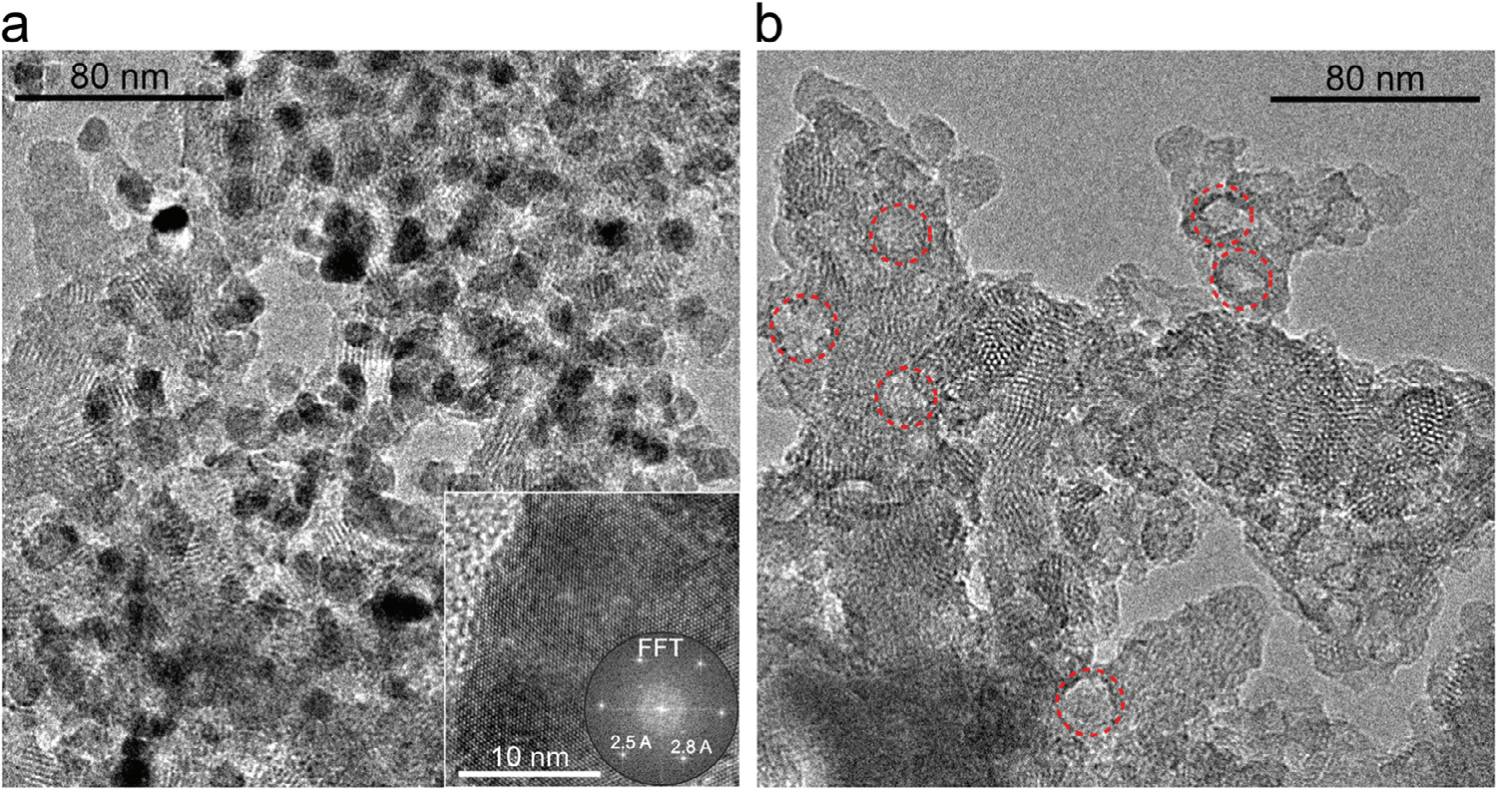
TEM analysis of a) ZnO@TpBz with dense regions attributed to ZnO NP as shown by FFT in the inset and b) hTpBz with absence of the dark ZnO particles and secondary textural pores at the brighter spots exemplified by red dotted circles. Size estimation of ZnO NPs (ZnO@TpBz, ≈18 nm) and the induced mesopores (hTpBz, ≈17 nm) was performed via an image analysis software (ImageJ 1.54e).

Scanning electron microscopy (SEM) and transmission electron microscopy (TEM) were employed to characterize and compare the morphology of the pristine and the hCOF samples. SEM images of the (h)Tp‐COFs show agglomerated polycrystalline particles with sizes up to multiple µm (Figures S40–S42, Supporting Information). In contrast to the more needle‐like structures of the hierarchical counterpart, the pristine Tp‐COFs particles exhibit spherical shapes at the edges. TEM images gave further insights into the nanoscale architecture of hTpBz. Imaging ZnO@TpBz revealed dense spots with diameters up to 20 nm next to a bright solid compound (COF), verifying the incorporation of ZnO NPs (**Figure** **2**a). Fast fourier transformation (FFT) of the dense spots on these images reveal d‐spacings of 2.5 and 2.8 Å which is in line with the reflections at 2*θ* = 31.8° and 36.2° of zinc oxide (Figure 2a, inset). FFT on the brighter solid fringes showed a lattice spacing of 23 Å, consistent with the lattice parameters of the TpBz framework (Figure S35, Supporting Information). The absence of the dark regions in TEM analysis of the acetic acid treated compound hTpBz demonstrates the successful removal of ZnO, while the remaining material shows hollow regions with diameters up to 20 nm, which are attributed to secondary pores exposed by template‐etching (Figure 2b, red circles). These results are nearly identical to the TEM images of the follow‐up candidates with larger pore size, i.e. TpTPD and TpQPD (Figures S36 and S37, Supporting Information).

**Figure 3:**
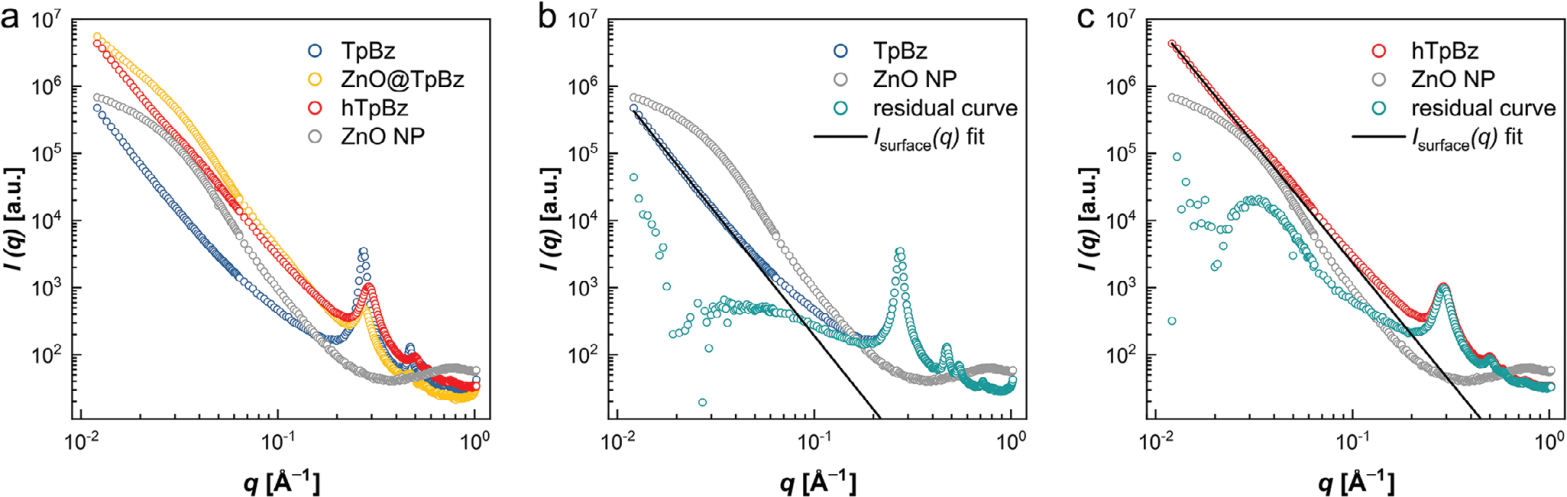
a) SAXS profiles of the (h)TpBz system in double‐logarithmic representation, with the TpBz (blue), ZnO@TpBz (yellow), hTpBz (red), and the ZnO NP suspension (gray). SAXS profiles of b) TpBz and c) hTpBz and the residual curves (turquoise) resulting from subtracting the power law description of the low q (0.017 – 0.15 Å^−1^) scattering from experimental data.

To further investigate the hierarchically porous nature of the synthesized materials, small‐angle X‐ray scattering (SAXS) measurements were performed for all described systems and demonstrated with the (h)TpBz‐COFs referenced to the curves of the ZnO nanoparticle dispersion. SAXS has previously been employed to study porous materials for reliable surface area determination, although its application to COFs has been limited so far.^[^ ^22^ ^]^ As shown in **Figure** **3**a, the SAXS profile of TpBz in the high *q* region (0.15 to 1.00 Å^−1^) exhibits the (100) and (110) reflections at 0.27 and 0.47 Å^−1^, respectively, in line with the recorded XRPD pattern. In the low *q* region (0.012 to 0.15 Å^−1^), the curve decays with a power law behavior according to

(1) $$I_{\mathrm{surface}}\left(q\right)=C\cdot q^{-\alpha }$$

arising from interfacial scattering, when differences in electron density exist within a two‐phase system.^[^ ^23^ ^]^ *C* is given by *C*  =  2π(*Δ* *ρ*)^2^ *S_V_*, where *Δρ* is the electron density difference between the two phases, or the contrast, and *S_V_* denotes the specific surface area of the sample. Note that the scattering intensity was measured in arbitrary units (see SI), meaning that *S_V_* could not be determined from the data analysis using Equation (1). According to Porod,^[^ ^23a^ ^]^ the power law exponent *α* exhibits a value of 4 for defined shape and sharp electron density discontinuity at the interface. However, *α* values between 3 and 4 were also found for diffuse interface profiles, e.g. due to surface roughness.^[^ ^23b,c^ ^]^ Fitting Equation (1) to the experimental data of TpBz yields an exponent *α* of 3.7 and a constant *C* = 0.024. Subtracting this scattering contribution, i.e. *I*_surface_(*q*), from the TpBz data, there is in fact no signal in the low *q*‐part of the residual curve (Figure 3b), indicating no secondary structure in this *q*‐region is present in the pristine TpBz. When looking at the scattering data of ZnO@TpBz, a reduction in intensity is observed in the crystal pattern at high *q*, while a significant increase in intensity can be observed at low *q*. Here, the power law behavior according to Equation (1) is superimposed with the scattering contribution of the ZnO NP, which becomes particularly evident in the region around *q* ≈ 0.032 Å^−1^ and aligns closely with the scattering curve of the ZnO NP dispersion, dominated by the form factor of the polydisperse and spherical ZnO NP.^[^ ^24^ ^]^ These observations are in good agreement with the form factor of ThO_2_ and UO_2_ NPs in the SAXS pattern, if embedded in COF‐5, as demonstrated by Moreau and co‐workers in their study analyzing the nanoparticle size distribution in a COF via SAXS.^[^ ^25^ ^]^ This similarity as well as the increased surface scattering at low *q* apparent in the ZnO@TpBz data prove the presence of ZnO NPs embedded in TpBz. Note that even though the data were not measured on absolute scale, the relative changes in scattering intensity should be reliable as the same thickness was used for all powder samples. Considering then the SAXS profile of hTpBz after acetic acid treatment, the scattering contribution of the ZnO NPs, as confirmed by XRPD combined with ICP‐OES analysis, is absent. In addition to the characteristic COF reflections at high *q*, the hTpBz data also reveal high scattering intensity almost following a power law behavior at low *q*. When describing this range of the hTpBz data with Equation (1), an exponent *α* of 3.6 and a constant *C* = 0.65 are obtained, indicating an increase of the specific surface area *S_V_*. Remarkably, the scattering contribution of the induced secondary pores, which is much weaker due to the much lower scattering contrast *Δρ* compared to the material with ZnO NPs, can be extracted by subtracting the surface scattering *I*_surface_(*q*) from the hTpBz data. The obtained residual curve in Figure 3c shows a broad maximum at low *q* similar to the scattering profile of the ZnO NP dispersion and this can indeed be attributed to scattering by the induced secondary pores, demonstrating the hierarchically porous nature of the synthesized hTpBz COF. The small shift of the maximum to higher *q*‐values compared to the ZnO NPs data indicates a minor (and expected) pore shrinkage upon removal of the template. These findings are in line with the template (≈18 nm) and void (≈17 nm) diameters extracted by a systematic TEM image analysis (see Supporting Information for details) and coincide with the fitted diameter (18 nm) obtained from the Guinier analysis for the pure ZnO NP suspension (Figure S18, Supporting Information).

### 2.2 Adsorption Experiments

**Figure 4:**
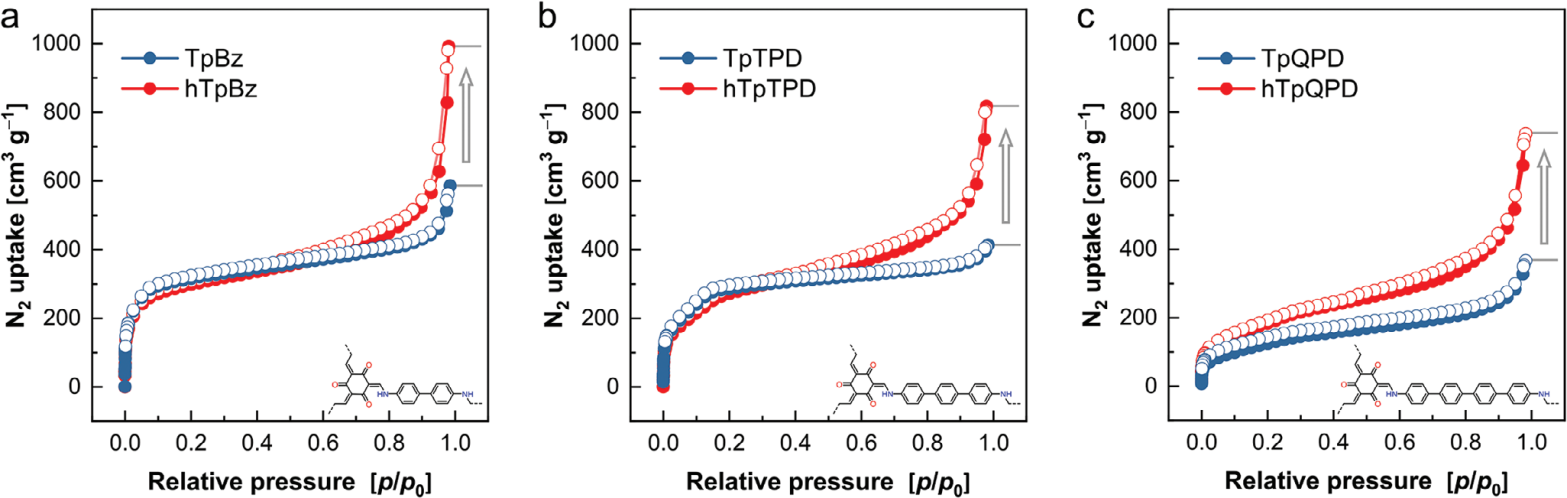
N_2_ adsorption isotherms of non‐hierarchically and hierarchically porous a) TpBz, b) TpTPD, and c) TpQPD systems.

Nitrogen gas adsorption experiments were conducted to analyze porosity and adsorption behavior of the pristine samples and the hTp‐COFs. Nitrogen adsorption isotherms of TpBz and hTpBz resemble type I(b) isotherms, indicating the presence of narrow mesopores in the range up to 2.5 nm (**Figure** **4**a).^[^ ^26^ ^]^ Toward high relative pressures with *P*∙*P_0_* ^−1^ between 0.8 and 1.0, the isotherm of hTpBz shows a significant increase in nitrogen uptake signaling the presence of additional larger mesopores. Furthermore, the total pore volume of hTpBz (1.54 cm^3^ g^−1^) is considerably higher than that of non‐hierarchically porous TpBz (0.91 cm^3^ g^−1^) (Figure 4a, **Table** **1**). In addition, calculation of the pore size distribution (PSD) results in pore sizes of 1.98 nm for both (h)TpBz systems and suggests that there is no change of the intrinsic structural porosity of the COF (Figure S19, Supporting Information). In the following, the structural pores of the COFs are classified as mesopores according to the IUPAC terminology (2– 50 nm), although they are being close to structural micropores (e.g. pore size of TpBz_(theoretical)_: 2.88 nm, TpBz_(PSD)_: 1.98 nm). The increase in overall total pore volume indicates the presence of pores with larger pore size and can here be attributed to the template‐induced textural mesopores. The comparably lower N_2_ uptake close to the pore filling step at 2 nm for hTpBz shown in the PSD stems from a decreased ratio of small structural mesopores of the COF vs. larger induced textural pores of the template. The non‐destructive effect on the framework's integrity of this templating approach is further supported by unaltered Brunauer‐Emmett‐Teller (BET) surface areas of TpBz (1178 m^2^ g^−1^) and its hierarchically porous counterpart (hTpBz: 1092 m^2^ g^−1^).

**Table 1:** Total pore volume (TPV), pore size and BET surface area (BET SA) of the *β*‐ketoenamine‐based COF series as well as the presented imine‐linked COF extracted from N_2_ gas adsorption experiments.

	TpBz	hTpBz	TpTPD	hTpTPD	TpQPD	hTpQPD	TAPB‐MeOTP	hTAPB‐MeOTP
TPV [cm^3^∙g^−1^]	0.91	1.54	0.64	1.3	0.57	1.1	0.98	2.1
Pore Size [nm]	1.98	1.98	2.52	2.61	2.75	2.75	3.20	3.10
BET SA [m^2^∙g^−1^]	1178	1092	810	727	466	687	731	1400

The generality of this template‐induced approach is further demonstrated by N_2_ adsorption experiments for the larger‐pore, *β*‐ketoenamine‐based (h)TpTPD and (h)TpQPD systems. The results are consistent with the (h)TpBz system (Figure 4b,c), except with a less pronounced pore filling step in the low pressure region (*P*∙*P_0_* ^−1^ ≈0.05) for (h)TpQPD (Figure 4c). However, a significant increase in N_2_ uptake at *P*∙*P_0_* ^−1^ ≈0.90 for both systems and an increase in total pore volume on going from the pristine samples (TpTPD: 0.64 cm^3^ g^−1^, TpQPD: 0.57 cm^3^ g^−1^) to the hierarchical COFs (hTpTPD: 1.3 cm^3^ g^−1^, hTpQPD: 1.1 cm^3^ g^−1^) can be clearly observed. The comparably steeper progression of the isotherms at moderate *P*∙*P_0_* ^−1^ of all hCOFs over the pristine counterparts is an additional indication for the presence of textural mesopores throughout the hierarchically porous materials. The calculated PSD for TpTPD and hTpTPD furnishes a single pore size of ≈2.51 ± 0.1 nm (Figure S22, Supporting Information), whereas the PSDs for the (h)TpQPD system shows a broad distribution for both samples which is centered at ≈2.75 nm (Figure S25, Supporting Information). The BET surface areas of TpTPD (810 m^2^ g^−1^) and hTpTPD (727 m^2^∙g^−1^) as well as TpQPD (466 m^2^ g^−1^) and hTpQPD (687 m^2^ g^−1^) are similar for the pristine and templated samples and hence additionally support the non‐destructive conditions of the reported approach. In line with the *β*‐ketoenamine‐based COF series, N_2_ adsorption isotherms of imine‐linked TAPB‐MeOTP and hTAPB‐MeOTP show similar results with a significantly increased N_2_ uptake for the hCOF in comparison to the non‐hierarchically porous counterpart (Figure S28, Supporting Information), further illustrating the generality of the reported method. What stands out in the isotherm of the imine COF hTAPB‐MeOTP is a hysteresis loop, which is not present in its pristine counterpart, typically suggesting the existence of ink‐bottle‐shaped pores or other network percolation effects, and could originate from the acetic acid treatment.^[^ ^27^ ^]^ Besides, water and acetonitrile vapor adsorption experiments were conducted to test the superior adsorption capacity of hierarchically porous hTpBz. The isotherms at 298 and 288 K for water and at 300 K for acetonitrile show the same trends observed for nitrogen adsorption, with a lower uptake at the pore filling step for the hTpBz and an increased overall uptake of vapor, further demonstrating the transferability of the porosity effect to different adsorbates (Figures S32–S34, Supporting Information).

### 2.3 Diffusivity Study

After successful structure and porosity characterization of the hTp‐COF systems, the influence of the altered pore size distribution on the diffusivity of hTpBz was investigated through pulsed field gradient (PFG) NMR, which tracks molecular motion and transport ranging from nanometers to hundreds of micrometers.^[^ ^28^ ^]^ As discussed in a previous study, acetonitrile (MeCN) was chosen to probe its self‐diffusion in the pore system of (h)TpBz, due to its frequent usage as organic solvent in syntheses and satisfactorily long T_2_ relaxation times.^[^ ^29^ ^]^ TpBz and hTpBz were loaded by condensation of MeCN vapor (Supporting Information for details) into the pores. This approach preferentially adsorbs the liquid in small (micro/meso) pores of the particles and thus reduces intensity contributions of extraparticulate liquid during NMR diffusion experiments.^[^ ^29^ ^]^ Analysis of the NMR signal decay vs. the gradient *B* enables the extraction of diffusion coefficients (*D*), which provide information about the molecular mobility within the sample as described by the Stejskal‐Tanner equation (Equation (2)).^[^ ^30^ ^]^ In this equation, the gradient factor *B* is defined by the gyromagnetic ratio (*γ*) of the observed nuclei gradient field strength (*g*), the gradient pulse duration (*δ*) and the diffusion time (*Δ*).

(2) $$I=I_{0}\;exp\left[-D\gamma^{2}g^{2}\delta^{2}\left(\Delta -\frac{\delta }{3}\right)\right]=I_{0}e^{\left[-DB\right]}$$

**Figure 5:**
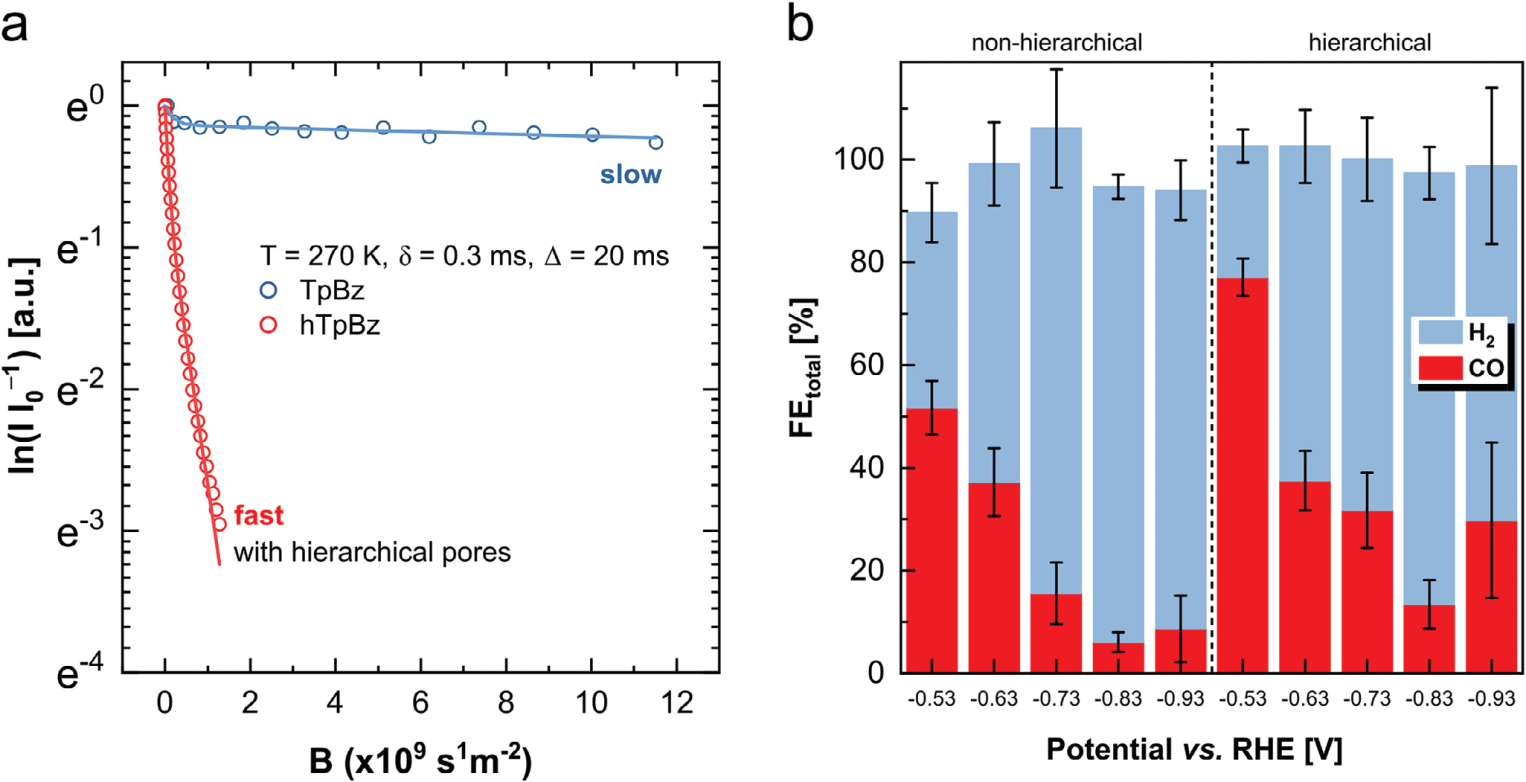
a) PFG‐NMR spin‐echo attenuation for MeCN loaded TpBz and hTpBz. Lines represent fits with a bi‐exponential model. b) Faradaic efficiencies (FE) of the CoTPP loaded (h)TpBz COFs at different potentials.

A direct comparison of PFG‐NMR spin‐echo attenuation for the same measurement parameters shows a much steeper decrease in signal intensity for hTpBz compared to TpBz, indicating faster diffusion, according to Equation (2). The use of a well‐established bi‐exponential model (**Figure** **5**a, see Supporting Information) to separate MeCN molecules which experienced gas‐liquid exchange (first exponential) from those remaining within the liquid (second exponential) during the diffusion experiment yields diffusion coefficients for liquid MeCN in the pores of 7.9 × 10^−12^ and 2.0 × 10^−9^ m^2^ s^−1^ for TpBz and hTpBz, respectively.

Since the isotropic diffusion radii (µm range, Table S3, Supporting Information) exceed the crystallite dimensions (≈30 nm, Figure S35, Supporting Information) for both samples, the determined diffusivities represent effective (intercrystallite) diffusion coefficients. The rapid interchange between filled, structural meso‐ and larger template‐induced mesopores in hTpBz results in effectively obtaining an averaged diffusion coefficient that is three orders of magnitude higher than in the predominantly structural porosity of TpBz due to the high diffusivity in the additional larger diameter pores. Thus, the effective diffusivity in hTpBz is very close to the self‐diffusion of acetonitrile in the bulk liquid at T = 270 K (2.6 × 10^−9^ m^2^ s^−1^), underlining the significant impact of the hierarchical structure in this sample on the molecular transport properties.

To probe the relevance of this improved mass transport in catalysis, an electrochemical carbon dioxide reduction (CO_2_R) study with (h)TpBz loaded with CoTPP was conducted. CoTPP is a well‐studied porphyrin based molecular catalyst for electrochemical CO_2_R with a good selectivity for CO production.^[^ ^31^ ^]^ The reproducibility of the loading method was ensured by ICP‐OES of three independent CoTPP@COF samples for TpBz and hTpBz, respectively (Supporting Information for details, Table S2, Supporting Information). Structural and morphological analysis via XRPD and SEM imaging reveal an essentially unchanged structure and morphology of the CoTPP free and the loaded (h)TpBz samples (Figures S13, S40, and S44, Supporting Information). For the electroreduction experiments, the heterogeneous COF catalysts were deposited on carbon paper substrates and subjected to an H‐cell set‐up under continuous CO_2_ flow (aq. KHCO_3_, 0.5 m, pH 7.3). Online gas chromatography (GC) analysis at five different potentials shows a superior Faradaic efficiency for CO in hierarchical CoTPP@hTpBz compared to its counterpart (Figure 5b). Further, the initial partial current density for CO (J_CO_) at the first GC injection, normalized by electrode surface area, demonstrates a more negative and thus larger current density for the hierarchically porous COF catalyst at −0.93 V vs. reversible hydrogen electrode (RHE) compared to CoTPP@TpBz (Figure S53, Supporting Information). At more negative current densities gaseous products form faster at the electrode surface, thus the catalysis becomes dependent on diffusion limitations of the system. The superior diffusivity of hTpBz, as demonstrated by PFG NMR, could be linked to improved molecular transport of the reactants and products (CO_2_, H_2_O, H_2_, CO etc.) in the hierarchical COF catalyst at more negative potentials (Figure S53, Supporting Information). We observe a nearly bulk‐like mobility of acetonitrile in the hierarchically porous hTpBz, which stems from an increased pore volume of the larger mesopores induced by the templating method. We hypothesize that the majority of electrolyte, reactants and products during catalysis are located in the large mesopores. These molecules show bulk‐like mobility and exchange rapidly with smaller, structural mesopores in the hCOF, leading to a superior mass transport throughout the material, including at reactive sites. This results in a positive impact of the template‐induced hierarchical porosity on the overall catalytic performance of the system.

## 3 Conclusion

In summary, we present a new, synthetically straightforward and general template‐induced approach to introduce hierarchical meso‐/mesoporosity through commercially available ZnO NPs in a series of *β*‐ketoenamine‐linked COFs and an imine‐based COF. To provide deeper insights into the hierarchical porosity of hCOFs, a multimodal combination of advanced characterization techniques such as SAXS and PFG NMR were utilized to analyze the template‐induced secondary porosity of hTpBz as a representative of the studied hierarchically porous hCOFs. In comparison to previously reported templating protocols based on PS nanoparticles, our method offers – besides incorporation of pores in the mesoporous regime (<50 nm) – a robust and transferable method applicable to solvothermal conditions, which retains the framework's structural integrity. All synthesized hCOF samples exhibit superior total pore volumes compared to their pristine counterparts without showing any significant decrease in crystallinity or surface areas. SAXS data of hTpBz COF conclusively demonstrate the scattering contributions of the generated secondary pores, thus demonstrating the hierarchically porous nature of the material. Our study demonstrates that hierarchical porosity in catalysts affects both gas and vapor sorption behavior and helps to overcome inherent diffusion limitations. Thus, PFG NMR of acetonitrile in TpBz and hTpBz reveals an effective diffusivity for hTpBz which is close to the self‐diffusion of bulk acetonitrile and orders of magnitude higher compared to TpBz, which demonstrates enhanced, more bulk‐like diffusion in the hierarchically porous structure. Likewise, electrochemical CO_2_R highlights the effectiveness of the templating approach, showing an improved Faradaic efficiency for CO in hTpBz as compared to its non‐hierarchical analogue. The presented ZnO NP‐based solvothermal templating approach thus provides a facile, rational and general method for introducing hierarchical porosity in COFs in order to significantly improve their adsorption capacity, diffusivity, and catalytic activity.

## Conflict of Interest

The authors declare no conflict of interest.

## Author Contributions

F.H. led the project, interpreted the data and wrote the manuscript. L.G. performed PFG NMR measurements and interpretation. N.S. and T.S. performed SAXS measurements and interpretation. A.H. assisted with the synthesis during the project. B.V.L. and L.Y. supervised the research. All authors wrote and commented on the manuscript.

## Supporting information



Supporting Information

## Data Availability

The data that support the findings of this study are openly available in DaRUS at https://doi.org/10.18419/darus‐4732, reference number 4732.

## References

[1] Q. Lu , G. S. Hutchings , W. Yu , Y. Zhou , R. V. Forest , R. Tao , J. Rosen , B. T. Yonemoto , Z. Cao , H. Zheng , J. Q. Xiao , F. Jiao , J. G. Chen , Nat. Commun. 2015, 6, 6567. 25910892 10.1038/ncomms7567 PMC4382682

[2] G. Collins , M. Blömker , M. Osiak , J. D. Holmes , M. Bredol , C. O'Dwyer , Chem. Mater. 2013, 25, 4312.

[3] S. Chakraborty , Y. J. Colón , R. Q. Snurr , S. T. Nguyen , Chem. Sci. 2015, 6, 384. 28966764 10.1039/c4sc02502d PMC5586205

[4] J. Konishi , K. Fujita , S. Oiwa , K. Nakanishi , K. Hirao , Chem. Mater. 2008, 20, 2165.

[5] X.‐Y. Yang , L.‐H. Chen , Y. Li , J. C. Rooke , C. Sanchez , B.‐L. Su , Chem. Soc. Rev. 2017, 46, 481. 27906387 10.1039/c6cs00829a

[6] a) C. M. A. Parlett , K. Wilson , A. F. Lee , Chem. Soc. Rev. 2013, 42, 3876; 23139061 10.1039/c2cs35378d

[7] a) L. Grunenberg , G. Savasci , S. T. Emmerling , F. Heck , S. Bette , A. Cima Bergesch , C. Ochsenfeld , B. V. Lotsch , J. Am. Chem. Soc. 2023, 145, 13241; 37231627 10.1021/jacs.3c02572 PMC10288504

[8] E. Jin , K. Geng , K. H. Lee , W. Jiang , J. Li , Q. Jiang , S. Irle , D. Jiang , Angew. Chem., Int. Ed. 2020, 59, 12162. 10.1002/anie.202004728 32329936

[9] a) R.‐R. Liang , X. Zhao , Org. Chem. Front. 2018, 5, 3341;

[10] A. K. Mohammed , S. Usgaonkar , F. Kanheerampockil , S. Karak , A. Halder , M. Tharkar , M. Addicoat , T. G. Ajithkumar , R. Banerjee , J. Am. Chem. Soc. 2020, 142, 8252. 32279483 10.1021/jacs.0c00555

[11] a) X. Zhao , P. Pachfule , S. Li , T. Langenhahn , M. Ye , C. Schlesiger , S. Praetz , J. Schmidt , A. Thomas , J. Am. Chem. Soc. 2019, 141, 6623; 30916950 10.1021/jacs.9b01226

[12] R. J. Kern , A. S. Kenyon , United States Patent, US3046245A, 1957.

[13] a) W. Li , H.‐X. Jiang , M.‐F. Cui , R. Wang , A.‐N. Tang , D.‐M. Kong , J. Hazard. Mater. 2022, 432, 128705; 35316634 10.1016/j.jhazmat.2022.128705

[14] J. Qiu , H. Wang , Y. Zhao , P. Guan , Z. Li , H. Zhang , H. Gao , S. Zhang , J. Wang , Green Chem. 2020, 22, 2605.

[15] J. van Embden , S. Gross , K. R. Kittilstved , E. D Gaspera , Chem. Rev. 2023, 123, 271. 36563316 10.1021/acs.chemrev.2c00456

[16] P. Pachfule , S. Kandmabeth , A. Mallick , R. Banerjee , Chem. Commun. 2015, 51, 11717. 10.1039/c5cc04130a 26104390

[17] a) R. L. Li , N. C. Flanders , A. M. Evans , W. Ji , I. Castano , L. X. Chen , N. C. Gianneschi , W. R. Dichtel , Chem. Sci. 2019, 10, 3796; 30996969 10.1039/c9sc00289h PMC6446964

[18] a) M. C. Daugherty , E. Vitaku , R. L. Li , A. M. Evans , A. D. Chavez , W. R. Dichtel , Chem. Commun. 2019, 55, 2680; 10.1039/c8cc08957d 30747178

[19] C. Stähler , L. Grunenberg , M. W. Terban , W. R. Browne , D. Doellerer , M. Kathan , M. Etter , B. V. Lotsch , B. L. Feringa , S. Krause , Chem. Sci. 2022, 13, 8253. 35919721 10.1039/d2sc02282f PMC9297439

[20] S. S. Kumar , P. Venkateswarlu , V. R. Rao , G. N. Rao , Int. Nano Lett. 2013, 3, 30.

[21] S. Kandambeth , A. Mallick , B. Lukose , M. V. Mane , T. Heine , R. Banerjee , J. Am. Chem. Soc. 2012, 134, 19524. 23153356 10.1021/ja308278w

[22] C. Schlumberger , C. Scherdel , M. Kriesten , P. Leicht , A. Keilbach , H. Ehmann , P. Kotnik , G. Reichenauer , M. Thommes , Microporous Mesoporous Mater. 2022, 329, 111554.

[23] a) G. Porod , Colloid. Polym. Sci. 1951, 124, 83;

[24] a) J. S. Pedersen , Adv. Colloid Interface Sci. 1997, 70, 171;

[25] L. M. Moreau , A. Herve , M. D. Straub , D. R. Russo , R. J. Abergel , S. Alayoglu , J. Arnold , A. Braun , G. J. P. Deblonde , Y. Liu , T. D. Lohrey , D. T. Olive , Y. Qiao , J. A. Rees , D. K. Shuh , S. J. Teat , C. H. Booth , S. G. Minasian , Chem. Sci. 2020, 11, 4648. 34122920 10.1039/c9sc06117g PMC8159168

[26] M. Thommes , K. Kaneko , A. V. Neimark , J. P. Olivier , F. Rodriguez‐Reinoso , J. Rouquerol , K. S. W. Sing , Pure Appl. Chem. 2015, 87, 1051.

[27] S. T. Emmerling , R. Schuldt , S. Bette , L. Yao , R. E. Dinnebier , J. Kästner , B. V. Lotsch , J. Am. Chem. Soc. 2021, 143, 15711. 34495671 10.1021/jacs.1c06518 PMC8485322

[28] J. Kärger , M. Avramovska , D. Freude , J. Haase , S. Hwang , R. Valiullin , Adsorption 2021, 27, 453.

[29] L. Grunenberg , C. Keßler , T. W. Teh , R. Schuldt , F. Heck , J. Kästner , J. Groß , N. Hansen , B. V. Lotsch , ACS Nano 2024, 18, 16091. 38860455 10.1021/acsnano.3c12167 PMC11210340

[30] J. E. Tanner , J. Chem. Phys. 1970, 52, 2523.

[31] X.‐M. Hu , M. H. Rønne , S. U. Pedersen , T. Skrydstrup , K. Daasbjerg , Angew. Chem., Int. Ed. 2017, 56, 6468. 10.1002/anie.201701104 28466962

