# Supplementary material for: Solvothermal Template‐Induced Hierarchical Porosity in Covalent Organic Frameworks: A Pathway to Enhanced Diffusivity
Source: Adv Mater. 2025 Jan 21;37(52):2415882. doi: 10.1002/adma.202415882 (PMC12747491; doi:10.1002/adma.202415882)
Supplement: Supplementary file 1 — Supporting Information [file ADMA-37-2415882-s001.docx]

**Supporting Information**

Solvothermal Template-Induced Hierarchical Porosity in Covalent Organic Frameworks: A Pathway to Enhanced Diffusivity

*Fabian Heck,^a,b,c^ Lars Grunenberg,^a,b^ Nadine Schnabel,^c,d^ Amelie Heilmaier,^a,b^ Thomas Sottmann,^c,d^ Liang Yao,^e,*^ Bettina V. Lotsch,^a,b,c,*^*

___________________________________________________________________________

^a^ Max Planck Institute for Solid State Research, Heisenbergstr. 1, 70569 Stuttgart, Germany

^b^ Department of Chemistry, Ludwig-Maximilians-Universität (LMU), Butenandtstr. 5-13, 81377 Munich, Germany

^c^ Collaborative Research Center 1333, Universität Stuttgart, Pfaffenwaldring 55, 70569 Stuttgart, Germany

^d^ Institute of Physical Chemistry, Pfaffenwaldring 55, 70569 Stuttgart, Germany

^e^ State Key Laboratory of Luminescent Materials and Devices, Institute of Polymer Optoelectronic Materials and Devices, Guangdong Basic Research Centre of Excellence for Energy and Information Polymer Materials, South China University of Technology, Guangzhou 510640, P. R. China

^*^ Email: b.lotsch@fkf.mpg.de, liangyao@scut.edu.cn

**Table of Contents**

**Methods and Instruments2**

General Methods2

Instruments2

Electrolyses6

**Syntheses8**

Synthesis of Tp Linker8

Tp-COF Synthesis9

Template-Induced Method: hTp-COFs9

Template-Induced Method: hTAPB-MeOTP COF10

Cobalt Loading of Tetraphenyl Porphyrin (TPP)10

Cobalt Tetraphenyl Porphyrin (CoTPP) Loading of COFs11

**Analytical Data12**

FT-IR Spectra12

XRPD Data14

SAXS Data20

Pulsed Field Gradient NMR (PFG-NMR)22

Nitrogen Gas Adsorption Experiments23

Vapor Adsorption Experiments29

TEM Images31

SEM Images32

Dynamic Light Scattering (DLS)35

Electrochemical Data36

**References40**

Methods and Instruments

**General Methods**

All reactions, unless otherwise noted, were performed with magnetic stirring in aluminum heating blocks. Reaction temperatures were electronically monitored. Chemicals were purchased from commercial sources and were used without further purification, as far as not stated otherwise. Dried glassware and standard laboratory equipment were used. All solvents were used as provided by the manufacturer, as far as not stated otherwise. Compound names are based on trivial names or were generated by PERKIN ELMER ChemDraw Professional version 20.0. The removal of solvent under reduced pressure was carried out on a standard rotary evaporator.

**Instruments**

**Dynamic light scattering (DLS)**

DLS measurements were carried out with a MALVERN ZETASIZER Nano ZS with a 4 mW HeNe laser (λ = 633 nm). The scattered light was detected in back-scattering geometry at an angle of 173°.

**Inductively coupled plasma optical emission spectroscopy (ICP-OES)**

ICP-OES measurements were conducted on a VARIAN VISTA PRO spectrometer. The characteristic wavelengths were separated with an Echelle-Polychromator (VARIAN, Darmstadt) and detected with a photomultiplier.

**Fourier transformed infrared spectroscopy (FTIR)**

FTIR spectra were recorded on a PERKIN ELMER UATR Two FTIR spectrometer equipped with an attenuated total reflection (ATR) measuring unit. The measurements were conducted with a resolution of 1 cm^−1^.

**Pulse Field Gradient Nuclear Magnetic Resonance Spectroscopy (PFG-NMR)**

Sample preparation: Dried COFs were exposed to a saturated MeCN vapor in air for two hours and the adsorbed amount of MeCN liquid was determined gravimetrically, leading to MeCN loadings of 0.33 g∙g^−1^ (TpBz) and 0.51 g∙g^−1^ (hTpBz). The MeCN loaded samples were quickly filled into NMR tubes, a PTFE-silicone plug was inserted and pushed into the tube until it reached a position below the spinner. Then the NMR tube was flame sealed and transferred to the spectrometer.

Diffusion experiments were performed in flame-sealed 5 mm NMR tubes on a Bruker Avance III 400 MHz spectrometer (*diff60* probe) at 270 K with a stimulated-echo sequence (*diffSte* program, Bruker TopSpin) without sample spinning. A variable temperature control unit supplied a chilled flow of N_2_ gas to ensure constant sample temperature during the experiment. To restrict the vapor volume to the temperature-controlled region below the sample spinner, a PTFE-silicone stopper was inserted into the NMR tube.^[1]^ Data processing was performed with the Bruker TopSpin 3.5 *Dynamics* module using manual integration and automated fitting of the integrated signal *I vs.* gradient *B* according to a biexponential model based on the Stejskal-Tanner equation (Eq. S1), where D_B_ represents an effective Diffusion coefficient of the liquid phase, while D_A_ contains major contributions of gas-liquid exchange.^[1-2]^

| $\frac{I}{I_{0}}=p_{A}\exp\left[ -BD_{A} \right]+p_{B}\exp\left[ -BD_{B} \right]$ | (Eq. S1) |
| --- | --- |

T_1_ relaxation times were determined using a standard inversion-recovery pulse sequence. T_2_ relaxation times were obtained by fitting the signal attenuation during a CPMG pulse sequence with appropriate delay times of d1 = 3-5×T_1_. For a typical measurement with COF, a linear sequence of gradient pulses (up to g_max_ = 9 T∙m^−1^) with a duration of δ = 0.3 ms (*opt* shape) and diffusion time Δ = 20 ms were used.

**X-Ray Powder diffraction (XRPD)**

XRPD patterns were recorded on a Stoe Stadi P diffractometer with a Co-/Cu-Kα_1_ source monochromatized with Ge(111) in a Debye-Scherrer geometry at room temperature (RT). The samples were sealed in ø1.0 mm glass No. 14 capillaries unless otherwise noted, and measured with spinning.

Structure modelling was initially performed with BIOVA Materials Studio 2017 (17.1.0.48. Copyright© 2016 Dassault Systèmes) software. The program TOPAS 6.0^[3]^ was used to refine the XRPD data of the COF samples. In order to obtain information on the inherent structural COF pore sizes, we performed Pawley refinements.^[4]^ The instrumental profile was described by the fundamental parameter approach implemented into the TOPAS software.^[5]^ For the Pawley fits we assumed trigonal and hexagonal cell metrics, even for the structure of TAPB-MeOTP, which is described in space group P1, *i.e.* we used the following constraints: *α* = *β* = 90°, *γ* = 120 °, a = b. In addition, we did not refine the c-axis lengths, as there are not 00l, hkl, h0l nor 0kl reflections apparent in the powder patterns. The resulting, refined cell parameter a can be taken as a measure for the inherent structural COF pore size, however, it must be noted that the effective inherent structural COF pore size is smaller due to the lateral extension of the COF linkers and nodes and due to randomized slipped stacking.^[6]^

Scanning electron microscopy (SEM)

SEM images were recorded on a Zeiss Merlin microscope at a voltage of 1.5 kV.

**Small-angle X-ray scattering (SAXS)**

SAXS measurements were performed on a Bruker AXS NanoStar X-ray diffractometer (Karlsruhe, Germany), using Cu *K_α_* radiation (λ = 1.542 Å) generated by a Kristalloflex 770 generator. The system uses a 100 μm point-collimated X-ray beam and a VÅNTEC 500 2D digital Mikrogap area detector. The samples were measured in a powder sample holder with a thickness of 1 mm trapped between two scotch tape stripes. The ZnO nanoparticle suspension (2.5wt% crystalline ZnO in isopropanol and propylene glycol, Sigma-Aldrich) was measured in a Mark capillary (Hilgenberg GmbH, glass No. 14) with an inner diameter of 0.6 mm and a glass thickness of 0.1 mm. Calibration for the scattering vector *q* was performed using silver behenate.^[7]^ Each sample was measured at two sample-to-detector distances (25 cm and 105 cm), the two resulting detector images were integrated azimuthally over the scattering angle 2*Θ* using the SAXS^TM^ software of Bruker and finally merged, to yield one dimensional scattering profiles $I\left( q \right)$ as a function of the scattering vector *q*, with *q* = 4π ∙ sin (*θ*) ∙ λ^−1^, to cover a total *q*-range between 0.01 – 1 Å^−1^.

**Nitrogen Gas Adsorption Experiments**

Nitrogen adsorption measurements were performed at 77 K on a QUANTACHROME INSTRUMENTS Autosorb iQ MP. The samples were degassed for 12 h at 120 °C under vacuum prior to the gas adsorption studies. BET analyses were conducted using a program written by Alexander M. Pütz (https://github.com/AlexanderPuetz) based on the software BETSI by Osterrieth and Fairen-Jimenez.^[8]^ The fitting ranges were selected to meet all four Rouquerol’s criteria and to minimize the pressure error of monolayer loading. Pore size distribution was determined from Nitrogen adsorption isotherms using the quenched solid density functional theory (QSDFT) on cylindrical pores in carbon model for nitrogen at 77 K.

**Supercritical carbon dioxide drying**

Supercritical carbon dioxide extraction was performed with a LEICA EM CPD300 critical point dryer and absolute methanol as exchange fluid.

**Vapor Adsorption Experiments**

Vapor sorption experiments were performed on a Quantachrome Instruments Autosorb iQ MP with water and acetonitrile at different temperatures. The samples were degassed for 12 h at 120 °C under vacuum prior to the adsorption studies. Values of the adsorbed amount VSTP [cm^3^∙g^−1^] were converted to gravimetric amount adsorbed per gram of material [g∙g^−1^] = V_STP_∙22414 ∙ 18.015^−1^.

**Transmission electron microscopy (TEM)**

TEM images were recorded with a PHILIPS CM30 ST (300 kV, LaB_6_ cathode). The samples were prepared dry onto a copper lacey carbon grid (Plano). TEM image analysis was conducted *via* image analysis software ImageJ 1.54e.

**Ultrasonic bath**

Sonication of the samples was carried out with an ELMASONIC S100 equipped with a high-performance sandwich transducer system.

**Electrolyses**

**Electrode preparation**

Catalyst inks were prepared with COF (2 mg), carbon black (Vulcan XC 72R, 1 mg∙mg^−1^ to COF), Nafion solution (1%, 2.5 µL∙mg^−1^ to COF), and EtOH (250 µL∙mg^−1^ to COF) by sonication and overnight magnetic stirring. 5% polytetrafluoroethylene (PTFE)-coated carbon paper (CP, Quintech TP-060-T5) was cut into a T shape with a catalyst-coating area (ca. 0.65 cm × 1.0 cm) and a non-coating area (ca. 0.40 cm × 1.0 cm). The resulting CP pieces were washed by sonication in ethanol (10 min) twice, followed by drying with compressed air. The catalyst ink was sonicated again and drop-casted onto the catalyst-coating areas of the CP pieces on both sides with 30 µL each (total COF loading amount: 0.24 mg). The drop-casted CP pieces were first dried at air, then under high vacuum and were connected to copper wires by sandwiching the non-coating areas and the copper wires with titanium foils and a custom-made polyetheretherketone (PEEK) electrode holder.

**Electrochemistry setup**

Electrochemical measurements and electrolysis were carried out with a Pine Research WaveDriver 200 EIS Bipotentiostat and a custom-designed gas-tight two-compartment H-cell. Anion exchange membrane (Fumatech fumasep FAB-PK-130) was pre-treated sequentially with 0.5 M NaCl aq, 0.5 M KOH aq, and 0.5 M KHCO_3_ aq, and then was assembled inside the H-cell. A three-electrode configuration was employed, with a Ag/AgCl electrode (porous glass/sat. KCl aq/AgCl/Ag) as a reference electrode and a Pt foil as a counter electrode. The working compartment of the H-cell was equipped with a reference electrode, a working electrode, a stir bar, a CO_2_ inlet tube, and a gas outlet tube. The counter compartment was equipped with a counter electrode and a leak valve. Both compartments were charged with 0.5 M KHCO_3_ aq. electrolyte (8.5 mL and 6.0 mL for the working and the counter compartment, respectively). The working compartment was subjected to a constant flow of CO_2_ (19.2 normal cubic centimeters per minute (nccm)) controlled by a Bronkhorst High-Tech F-201DV mass flow controller under magnetic stirring and the control of an overpressure valve (1150 mbar). Before performing any electrochemical experiment, the electrolyte was bubbled with CO_2_ for 10 min to saturate it with CO_2_ and to remove oxygen in the system, and the working electrodes were activated by cathodic scan cycles (0 V to −0.78 V *vs.* RHE, 20 mV∙s^−1^, 5 times). The electrolyte pH was 7.3 after being saturated with CO_2_. All experiments were carried out at ambient temperature. Cyclic voltammograms were recorded with a scan rate of 5 mV∙s^−1^.

Electrode potentials were converted to the reversible hydrogen electrode (RHE) scale using ERHE = *E*_Ag/AgCl_ + 0.059 × pH + 0.197 without *iR* compensation. To check the influence of *iR* drop, the uncompensated resistance (*R_u_*) was measured using electrochemical impedance spectroscopy (EIS) with an electronically equivalent circuit containing *R_u_* in a series to a parallel set of a constant phase element and a resistor. Fitting of curves at −0.58 V and −0.78 V vs RHE both showed *R_u_* = 4.5 Ω. Since the *iR* drop (< 0.04 V) is minor compared to the potential range applied in electrolyses (0.3 V), *iR* compensation has not been performed for our measurements.

**Gaseous product quantification**

Gas quantification has been conducted in a setup similar to that reported in previous work.^[9]^ Outflow gas was introduced into an autosampler of a Shimadzu Nexus GC-2030 gas chromatograph (GC) by intervals of 11.2 min, chromatographed with helium (99.999%, Air Liquide) as a carrier gas, and detected with a dielectric-barrier discharge ionization detector (BID).

The low headspace volume of the H-cell allows a steady-state response at the reactor outlet in the order of 1 min. The GC was calibrated with standard calibration gas mixtures containing CO, H_2_, and CH_4_ purchased from Air Liquide (4-point linear calibration, R^2^ > 0.999).

**Catalysis parameter calculation**

The faradaic efficiency (FE) for each product was calculated using the following equation:

$$\mathrm{FE}\boldsymbol{=}\frac{xPV}{RT_{r}}\boldsymbol{\cdot}\frac{\upsilon F}{I}$$

where *x* is the mole fraction of the product in the outflow gas measured by the GC, *P* is the pressure (1150 mbar) controlled by the back pressure controller, *V* is the volumetric gas flow rate referenced to volume at 0 °C controlled by the mass flow controller (19.2 mL∙min^−1^), *R* is the ideal gas constant, *T_r_* is the reference temperature (0 °C), *υ* is the number of electrons transferred (2 for CO and H_2_), *F* is the Faraday constant, and *I* is the total current measured by the potentiostat.

**Syntheses**

**Synthesis of Tp linker**

Tp linker was synthesized according to a modified standard literature procedure.^[10]^ Hexamethylenetetramine (12.9 g, 92.0 mmol, 2.21 eq.) and phloroglucinol (5.30 g, 41.6 mmol, 1.00 eq.) were dissolved in TFA (75 mL) and stirred at 100 °C for 2.5 h under argon gas atmosphere. Aqueous hydrochloric acid (37 mL, 3 M) was added *via* dropping funnel and after 1 h the reaction was allowed to cool down followed by further stirring at room temperature overnight. The reaction mixture was extracted with DCM (3 × 120 mL) as well as Brine (1 × 150 mL) and the solvent was removed *in vacuo*. The crude product was purified with hot ethanol (80 mL) to afford a pale-yellow solid (1.19 g, 5.66 mmol, 14%). The ^1^H NMR data coincides with the literature. ^1^H NMR (400 MHz, CDCl_3_): δ=14.12 (s, 3H, OH), 10.16 (s, 3H, CHO) ppm.^[11]^

**Tp-COF Synthesis**

Amine linker (benzidine: 13.2 mg, 71.4 μmol, 4,4''-diamino-*p*-terphenyl: 18.6 mg, 71.4 μmol, [1,1':4',1'':4'',1'''-Quaterphenyl]-4,4'''-diamine: 24 mg, 71.4 μmol, 1.50 eq.) was dissolved in DMF (3 mL) in a microwave vial, sealed, and heated to 90 °C. 1,3,5-Triformylphloroglucinol (10.0 mg, 47.6 μmol, 1.00 eq.) was dissolved in DMF (1.5 mL), sonicated, and added to microwave vial *via* a syringe pump (1.5 mL·h^−1^). The reaction mixture was stirred at 300 rpm for 24 h. The crude COF was washed with DMF, acetone, and methanol followed by purification *via* SOXHLET extraction. After drying with supercritical carbon dioxide, Tp-COFs were obtained as orange to yellow powders (TpBz: 20.4 mg, 99%, TpTPD: 25.3 mg, 97%, TpQPD: 30.6 mg, 97%).

**Template-Induced Method: hTp-COFs**

Amine linker (benzidine: 13.2 mg, 71.4 μmol, 4,4''-diamino-*p*-terphenyl: 18.6 mg, 71.4 μmol, [1,1':4',1'':4'',1'''-Quaterphenyl]-4,4'''-diamine: 24 mg, 71.4 μmol, 1.50 eq.) and ZnO NPs (2.5wt% crystalline ZnO suspension in isopropanol and propylene glycol purchased from Sigma Aldrich, 20 nm, 500 μL, 10.5 μL per μmol of 1,3,5-Triformylphloroglucinol) were suspended in DMF (3 mL) in a microwave vial, sealed, and heated to 90 °C. 1,3,5-Triformylphloroglucinol (10.0 mg, 47.6 μmol, 1.00 eq.) was dissolved in DMF (1.5 mL), sonicated, and added to microwave vial *via* a syringe pump (1.5 mL·h^−1^). The reaction mixture was stirred at 300 rpm for 24 h. The crude COF was washed with DMF, acetone, and methanol followed by purification *via* SOXHLET extraction. After drying with supercritical carbon dioxide, Tp-COFs were obtained as orange to yellow powders (ZnO@TpBz: 32.6 mg, ZnO@TpTPD: 38.0 mg, ZnO@TpQPD: 43.8 mg).

In the following step, the ZnO@Tp-COFs were suspended in acetic acid (5 mL, 6 m) and stirred (300 rpm) at room temperature for 4 h. The treated COF was thoroughly washed with water, acetone, and methanol, followed by purification *via* SOXHLET extraction. After drying with supercritical carbon dioxide, hTp-COFs were obtained as orange to yellow powders. The yields represented a loss of ~70wt% in line with the ICP-OES results (**Table S 1**).

Table S 1: Zn amount in wt% of the ZnO loaded Tp-COFs and the hTp-COFs after etching with acetic acid measured by ICP-OES.

| ICP-OES [wt% of Zn] | TpBz | TpTPD | TpQPD | TAPB-MeOTP |
| --- | --- | --- | --- | --- |
| ZnO loaded COF | 29.82 | 29.77 | 30.40 | 30.00 |
| after etching | 0.093 | 0.3495 | 0.4175 | 0.2863 |

**Template-Induced Method: hTAPB-MeOTP**

hTAPB-MeOTP was synthesized according to a modified literature procedure.^[12]^ 2,5-dimethoxyterephthalaldehyde (MeOTP, 16.7 mg, 85.8 μmol) and dichloromethane (0.35 mL) were placed in a vial and sonicated for 10 s. Acetonitrile (8.83 mL) was added and the resulting solution was stirred at room temperature until MeOTP was dissolved. To the linker solution 1,3,5-tris(4-aminophenyl)benzene (TAPB, 21.6 mg, 57.2 μmol, purity: 93%) was added and combined with the ZnO NP suspension (2.5wt% ZnO NP suspension in isopropanol and propylene glycol purchased from Sigma Aldrich, Ø = 20 nm, 620 μL, 10.8 μL per μmol TAPB). For the control with TAPB-MeOTP, propylene glycol (352 μL) and iPrOH (268 μL), the solvents of the purchased ZnO nanoparticle suspension, were utilized to exclude potential influence of the solvent on the COF crystallinity.

The vial was sealed and Sc(OTf)_3_ (7 mg, 14.2 μmol, 0.08 eq. per amine functional group in TAPB) in 2 mL acetonitrile was injected to the reaction tube within 5 min. The reaction was stirred (250 rpm) at room temperature for 20 hours. To collect the particle powder for, 1 M NaCl aq. solution (200 μL) was added to the vial to precipitate out the particles. The precipitates were collected with a filter paper, washed with acetone, dimethylformamide, chloroform and methanol, and subjected to SOXHLET extraction with methanol for 12 h. The product (ZnO@TAPB-MeOTP: 37.4 mg, TAPB-MeOTP: 30.0 mg, yield: 84%) was obtained after activation with supercritical CO_2_. In the following etching step, the ZnO@TAPB-MeOTP was suspended in acetic acid (6.2 mL, 6 m) and stirred (250 rpm) at room temperature for 4 h. The treated COF was thoroughly washed with water, acetone, and methanol, followed by purification *via* SOXHLET extraction. hTAPB-MeOTP (30.9 mg) was obtained after activation with supercritical CO_2_.

**Cobalt loading of Tetraphenyl Porphyrin**

Tetraphenyl porphyrin (TPP) (200 mg, 325 μmol, 1.00 eq.) and cobalt acetate (162 mg, 650 μmol, 2.00 eq.) were combined with DMF (12 mL) and the mixture was purged *via* a needle with argon for 10 min and a second purging without a needle. The reaction mixture was heated at 120 °C for 19 h. After cooling to room temperature, the solution was transferred into 40 mL water/ice mixture. The product was filtered, washed with methanol and dried *in vacuo* overnight. Cobalt(II) tetraphenyl porphyrin was obtained as a dark purple powder (169 mg, 77%) and analyzed with ICP-OES to confirm the successful loading of cobalt. (Co: 8.61wt%)

**Cobalt Tetraphenyl Porphyrin Loading of COFs**

A Cobalt(II) tetraphenyl porphyrin (CoTPP) stock solution was prepared with 2 mg mL^−1^ in CHCl_3_. The stock solution was added to (h)TpBz-COFs (5 mg mL^−1^), heated to 65 °C and stirred (300 rpm) for 4 h. Then loaded COFs were washed with small amounts of warm CHCl_3_ and methanol. After drying with supercritical carbon dioxide, CoTPP loaded (h)TpBz-COFs were obtained as orange powders.

To ensure a reproducible loading, three separate batches of (h)TpBz-COF were loaded with CoTPP and analyzed *via* ICP-OES.

Table S 2: Cobalt amount in wt% of the CoTPP loaded TpBz and hTpBz in three separate batches.

|  | 1 | 2 | 3 | Ø |
| --- | --- | --- | --- | --- |
| **CoTPP@TpBz** | 0.6535 | 0.6445 | 0.6405 | 0.64617 |
| **CoTPP@hTpBz** | 0.5650 | 0.6430 | 0.6650 | 0.62433 |

**Analytical Data**

**FT-IR Spectra**

Figure S 1: FT-IR ATR spectra comparison of TpTPD, ZnO@TpTPD and hTpTPD.

Figure S 2: FT-IR ATR spectra comparison of TpQPD, ZnO@TpQPD and hTpQPD.

Figure S 3: FT-IR ATR spectra comparison of TAPB-MeOTP, ZnO@TAPB-MeOTP and hTAPB-MeOTP.

**XRPD Data**

Figure S 4: XRPD comparison of different template loading amounts in TpBz with an optimum at 10.5 μL of ZnO NP suspension (2.5wt% ZnO NP suspension in isopropanol and propylene glycol purchased from Sigma Aldrich, Ø = 20 nm) per μmol of Tp linker. The amount of added ZnO suspension is referenced to the molar mass of the Tp linker for the reaction. Measured with Cu Kα_1_ radiation.

The comparative analysis of XRPD data for different amounts of ZnO suspensions (2.5wt% ZnO NP suspension in isopropanol and propylene glycol purchased from Sigma Aldrich, Ø = 20 nm) was conducted by examining the relative peak intensities of the COF reflections as a proxy for crystallinity in various ZnO@TpBz samples. Notably, there was a significant enhancement in the intensity of the (100) plane reflection for the sample containing 10.5 μL of ZnO NP suspension per micromole of Tp (red). The lower loadings only show a weak intensity of the (100) plane reflection, where as in the higher loadings no (100) plane reflection was evident. It is important to consider that as the ZnO amount increases, the relative intensity of the COF reflections tends to decrease due to the reduced scattering contrast of the framework material. Nevertheless, the influence of varying amounts of isopropanol/propylene glycol mixture of the suspension in combination with the ZnO NPs on the COF formation has resulted in optimal conditions at a template suspension volume of 10.5 μL of ZnO NP suspension per micromole of Tp linker.

Figure S 5: XRPD comparison of TpBz *vs*. a simulated TpBz with eclipsed AA stacking and staggered AB stacking. Measured with Cu Kα_1_ radiation.

Figure S 6: Pawley refinement of TpBz with refined cell parameter *a* as a measure for the inherent structural (theoretical) COF pore size. Calculated pore size based on the position of the (100) plane reflection: 2.6 nm.

Figure S 7: XRPD comparison of TpTPD, ZnO@TpTPD and hTpTPD against a simulated TpTPD with eclipsed AA stacking and simulated ZnO. Measured with Cu Kα_1_ radiation.

Figure S 8: Pawley refinement of TpTPD with refined cell parameter *a* as a measure for the inherent structural (theoretical) COF pore size. Calculated pore size based on the position of the (100) plane reflection: 3.2 nm.

Figure S 9: XRPD comparison of TpQPD, ZnO@TpQPD and hTpQPD against a simulated TpQPD with eclipsed AA stacking and simulated ZnO. Measured with Cu Kα_1_ radiation.

Figure S 10: Pawley refinement of TpQPD with refined cell parameter *a* as a measure for the inherent structural (theoretical) COF pore size. Calculated pore size based on the position of the (100) plane reflection: 3.8 nm.

Figure S 11: XRPD comparison of TAPB-MeOTP, ZnO@ TAPB-MeOTP and h TAPB-MeOTP against simulated XRPD of TAPB-MeOTP with eclipsed AA stacking and simulated ZnO. Measured with Measured with Cu Kα_1_ radiation.

Figure S 12: Pawley refinement of TAPB-MeOTP with refined cell parameter *a* as a measure for the inherent structural (theoretical) COF pore size. Calculated pore size based on the position of the (100) plane reflection: 3.2 nm.

Figure S 13: XRPD comparison of CoTPP loaded TpBz and hTpBz. Measured with Measured with Cu Kα_1_ radiation.

**SAXS Data**

**
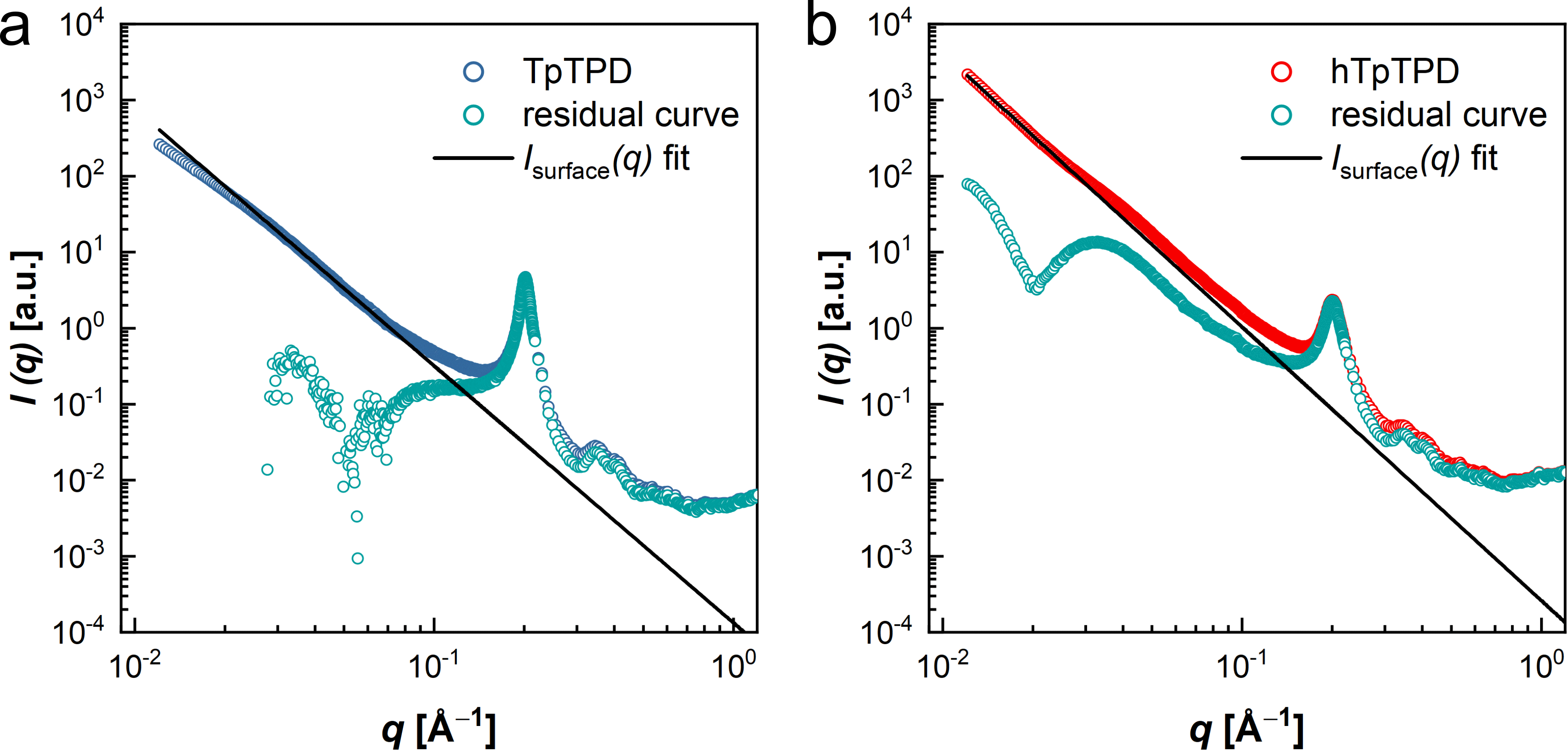
**

Figure S 14: SAXS profiles of the (h)TpTPD system in double-logarithmic representation, with the (a) TpTPD (blue) and (b) hTpTPD (red). The residual curves (turquoise) resulting from subtracting the power law description of the low *q* (0.017 – 0.15 Å^−1^) scattering from experimental data. In addition to the characteristic COF peaks at high *q* (>0.16 Å−1), both samples reveal high scattering intensity almost following a power law behaviour at low *q* (<0.16 Å−1). When describing this range with *I*_surface_(*q*) (Eq.1) and subtracting the surface scattering from the SAXS data (residual curve), hTpTPD shows a broad maximum in the region of *q* ≈ 0.032 Å^−1^ and can indeed be attributed to scattering by the induced secondary pores. These findings are perfectly in line with the data obtained from (h)TpBz and demonstrates the successful incorporation of the hierarchical porosity.

**
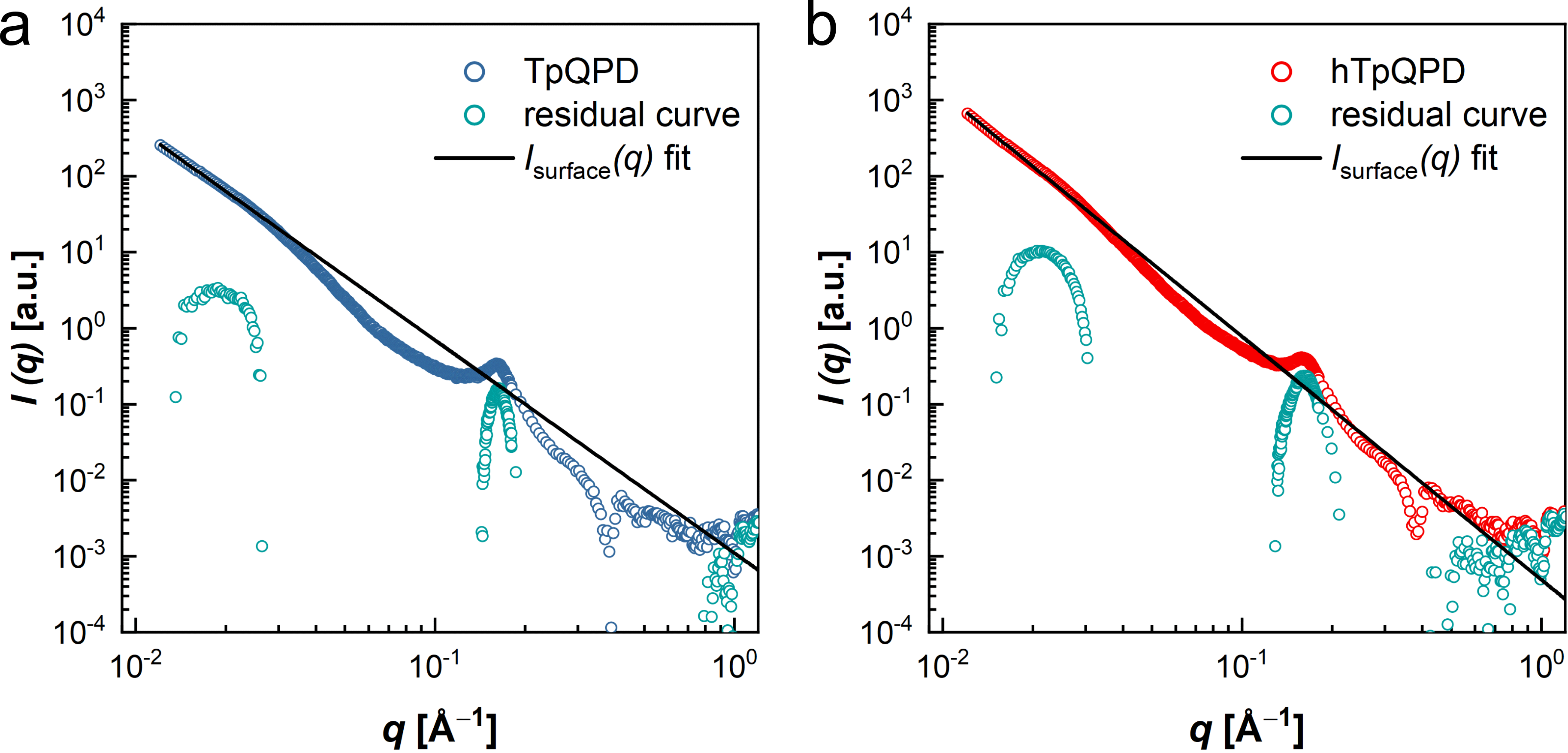
**

Figure S 15: SAXS profiles of the (h)TpQPD system in double-logarithmic representation, with the (a) TpQPD (blue) and (b) hTpQPD (red). The residual curves (turquoise) resulting from subtracting the power law description of the low *q* (0.017 – 0.13 Å^−1^) scattering from experimental data. Subtracting the Porod surface scattering of (h)TpQPD resulted in loss of data in the mesoscopic regime leading to a discontinuity of the residual curve and, therefore, do not show the expected maximum for the induced secondary pore. This originates from the dependency of the Porod law on the factor *α* - a parameter indicative of the surface roughness - which also varies with the detected length scale. However, in the case of (h)TpQPD, the observed discontinuity of the residual curve could be addressed by extending measurements to lower *q* values, thereby enhancing the Porod fit of the surface scattering.


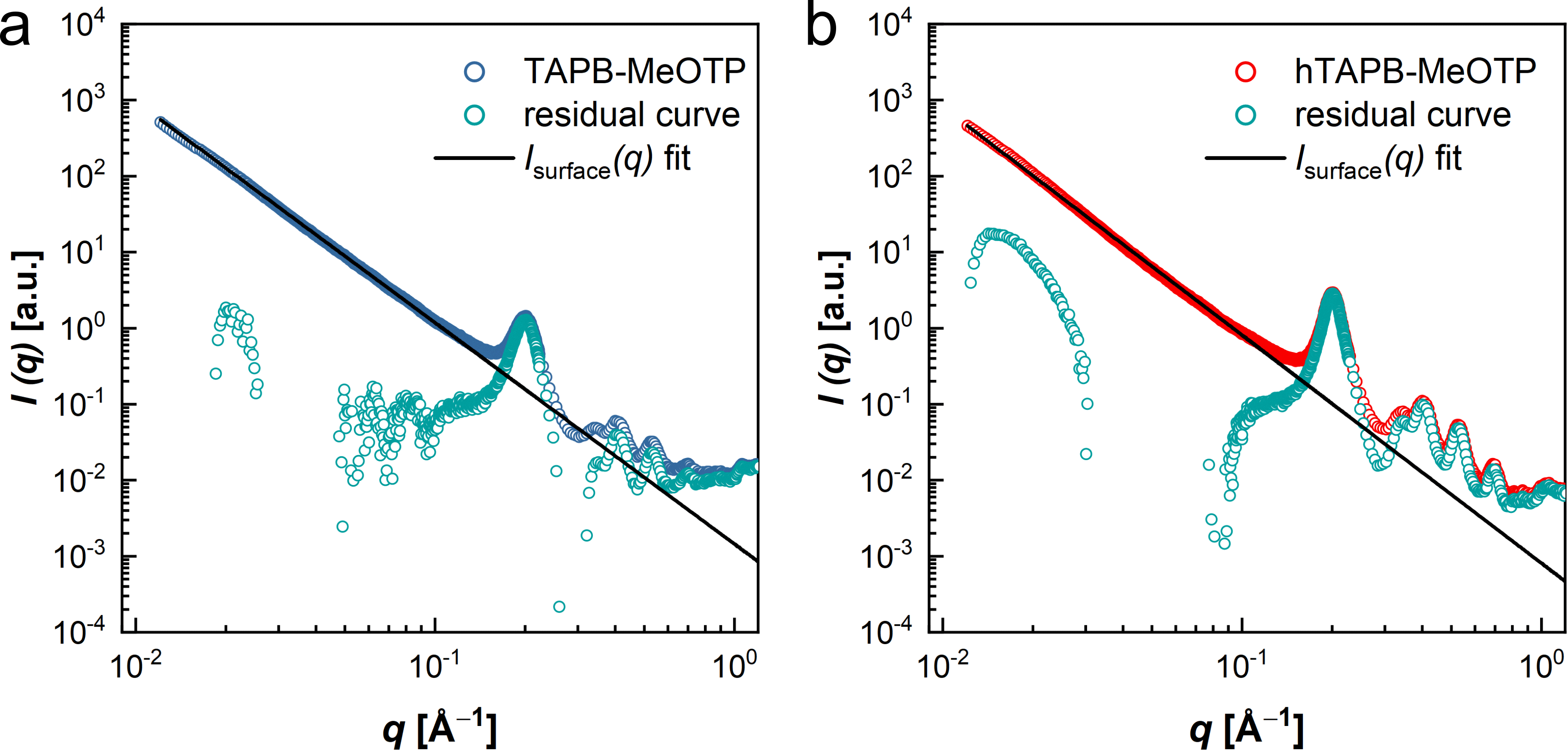


Figure S 16: SAXS profiles of the (h)TAPB-MeOTP system in double-logarithmic representation, with the (a) TAPB-MeOTP (blue) and (b) hTAPB-MeOTP (red). The residual curves (turquoise) resulting from subtracting the power law description of the low q (0.017 – 0.15 Å^−1^) scattering from experimental data. In line with the measurements for (h)TpQPD, the discontinuity resulted from the subtraction of the modelled Porod surface scattering. Therefore, the expected maximum for the induced secondary pores cannot be observed in this plot.

Figure S 17: Form factor of polydisperse spheres (PY structure factor) for the purchased ZnO NP suspension (2.5wt% in propylene glycol and isopropanol).

Figure S 18: Guinier analysis of the purchased ZnO NP suspension (2.5wt% in propylene glycol and isopropanol) with an obtained radius of 90 Å.

**Pulsed field gradient NMR (PFG-NMR)**

Table S 3: T_1_/T_2_ relaxation times and diffusion coefficients, and isotropic diffusion radii of acetonitrile (MeCN) compared to acetonitrile loaded on TpBz and hTpBz at T = 270 K and Δ = 20 ms.

|  | **T_1_ [s]** | **T_2_ [ms]** | **D [m²s^−1^]** | **r [µm]** |
| --- | --- | --- | --- | --- |
| MeCN^[13]^ | 12.3 | 730 | 2.63 × 10^−9^ ± 3 × 10^−11^ (D_B_) | - |
| TpBz + MeCN | 1.1 | 0.45 | 7.9 × 10^−12^ ± 2 × 10^−13^ (D_B_) | 0.6 |
| hTpBz + MeCN | 0.3 | 1.2 | 2.01 × 10^−9^ ± 2 × 10^−11^ (D_B_) | 8.9 |
|  |  |  |  |  |

**Nitrogen Gas Adsorption Experiments**

Figure S 19: Pore size distribution (PSD) for TpBz and hTpBz.

Figure S 20: BET plot for TpBz.

Figure S 21: BET plot for hTpBz.

Figure S 22: Pore size distribution (PSD) for TpTPD and hTpTPD.

Figure S 23: BET plot for TpTPD.

Figure S 24: BET plot for hTpTPD.

Figure S 25: Pore size distribution (PSD) for TpQPD and hTpQPD.

Figure S 26: BET plot for hTpQPD.

Figure S 27: BET plot for hTpQPD.

Figure S 28: Nitrogen adsorption isotherms of TAPB-MeOTP and hTAPB-MeOTP.

Figure S 29: Pore size distribution (PSD) for TAPB-MeOTP and hTAPB-MeOTP.

Figure S 30: BET plot for TAPB-MeOTP.

Figure S 31: BET plot for hTAPB-MeOTP.

**Vapor Adsorption Experiments**

Figure S 32: Water vapor adsorption isotherms of TpBz and hTpBz at 298 K.

Figure S 33: Water vapor adsorption isotherms of TpBz and hTpBz at 288 K.

Figure S 34: Acetonitrile (ACN) vapor adsorption isotherms of TpBz and hTpBz at 300 K.

**TEM Images**


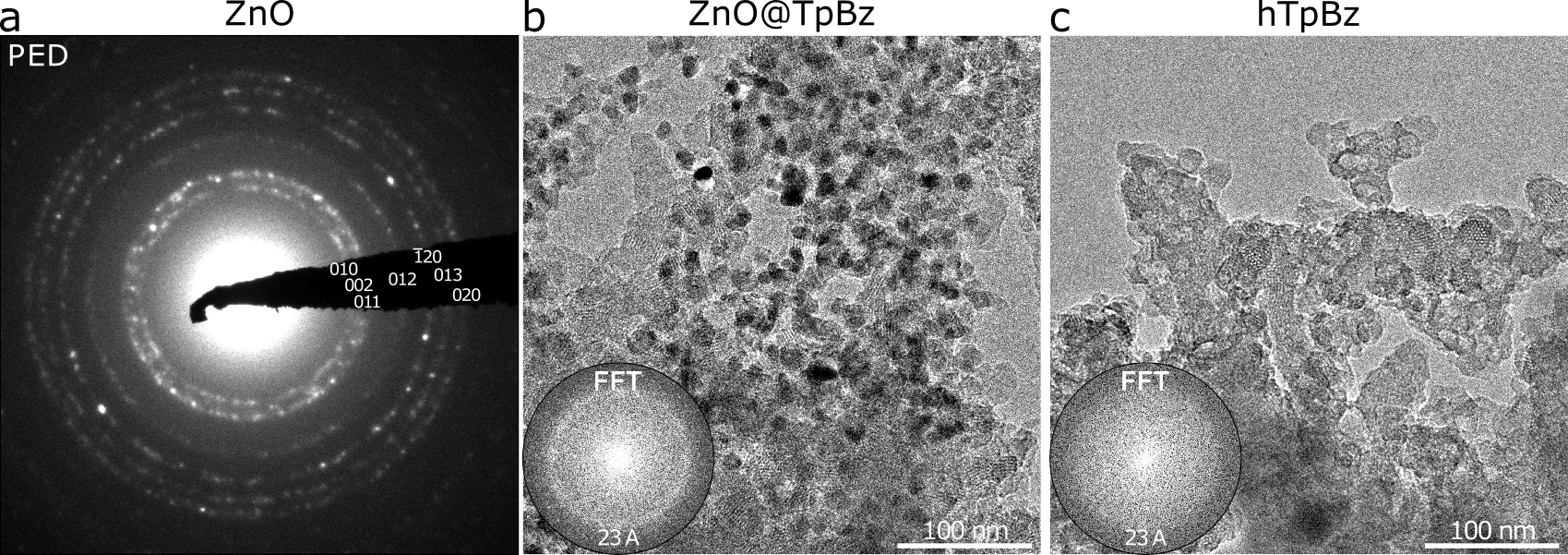


Figure S 35: a) Precession electron diffraction (PED) obtained within the region containing ZnO NPs and TEM images of b) ZnO@TpBz and c) hTpBz.


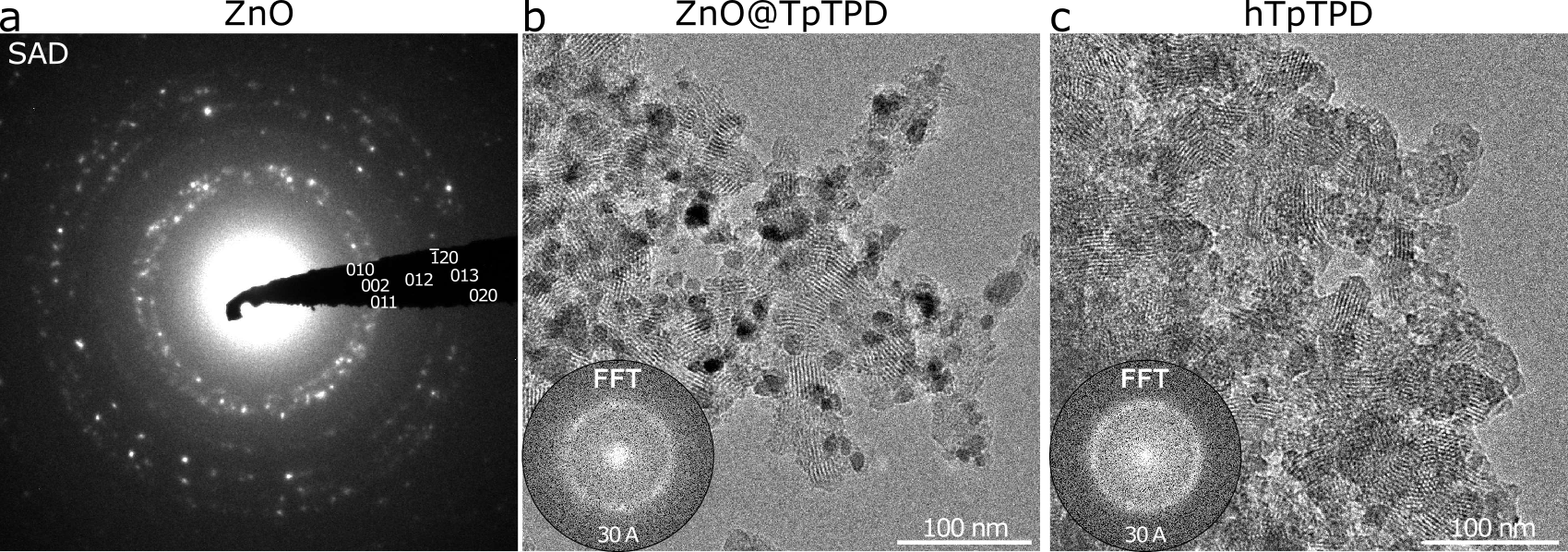


Figure S 36: a) Selected area electron diffraction (SAED) obtained within the region containing ZnO NPs and TEM images of b) ZnO@TpTPD and c) hTpTPD.


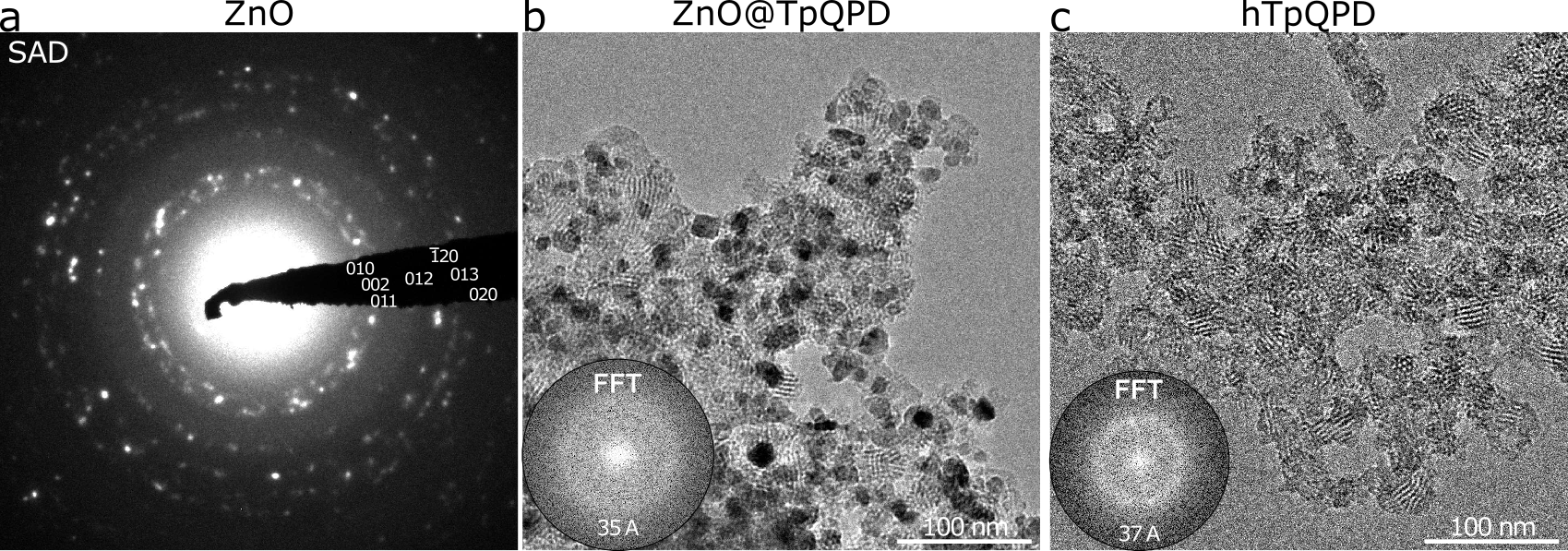


Figure S 37: a) Selected area electron diffraction (SAED) obtained within the region containing ZnO NPs and TEM images of b) ZnO@TpQPD and c) hTpQPD.


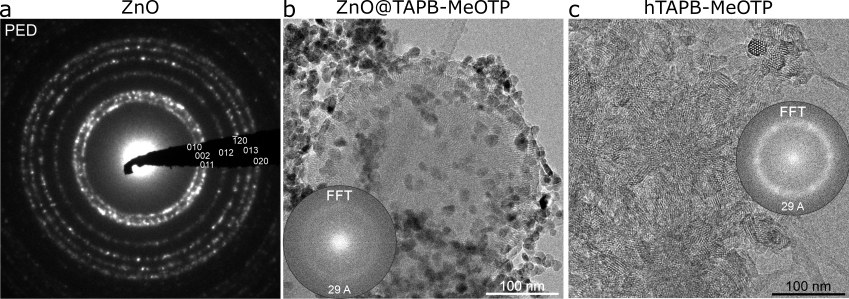


Figure S 38: a) Precession electron diffraction (PED) obtained within the region containing ZnO NPs and TEM images of b) ZnO@TAPB-MeOTP and c) hTAPB-MeOTP.

**
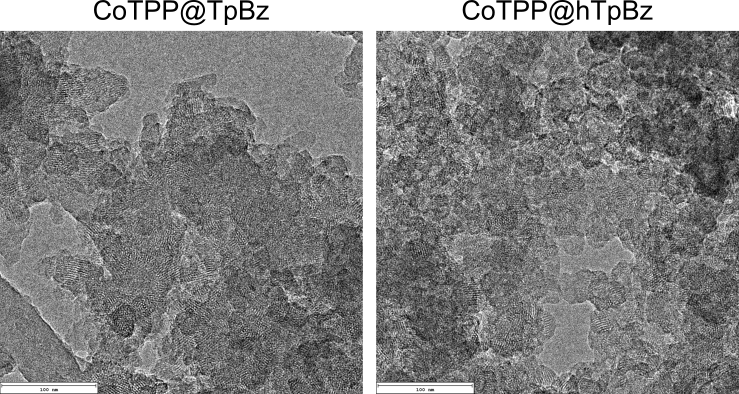
**

Figure S 39: TEM images of TpBz and hTpBz after loading with CoTPP (0.1 mg mL^−1^).

**SEM Images**


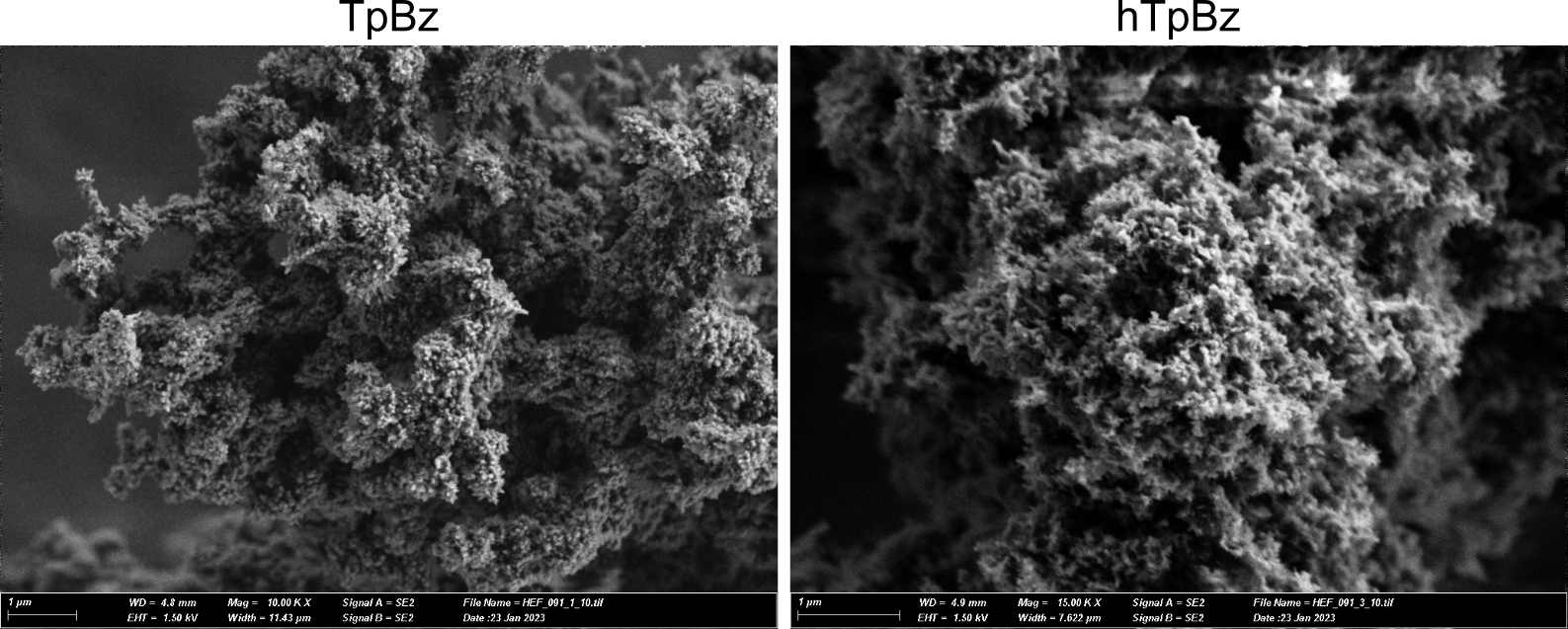


Figure S 40: SEM images of TpBz and hTpBz.


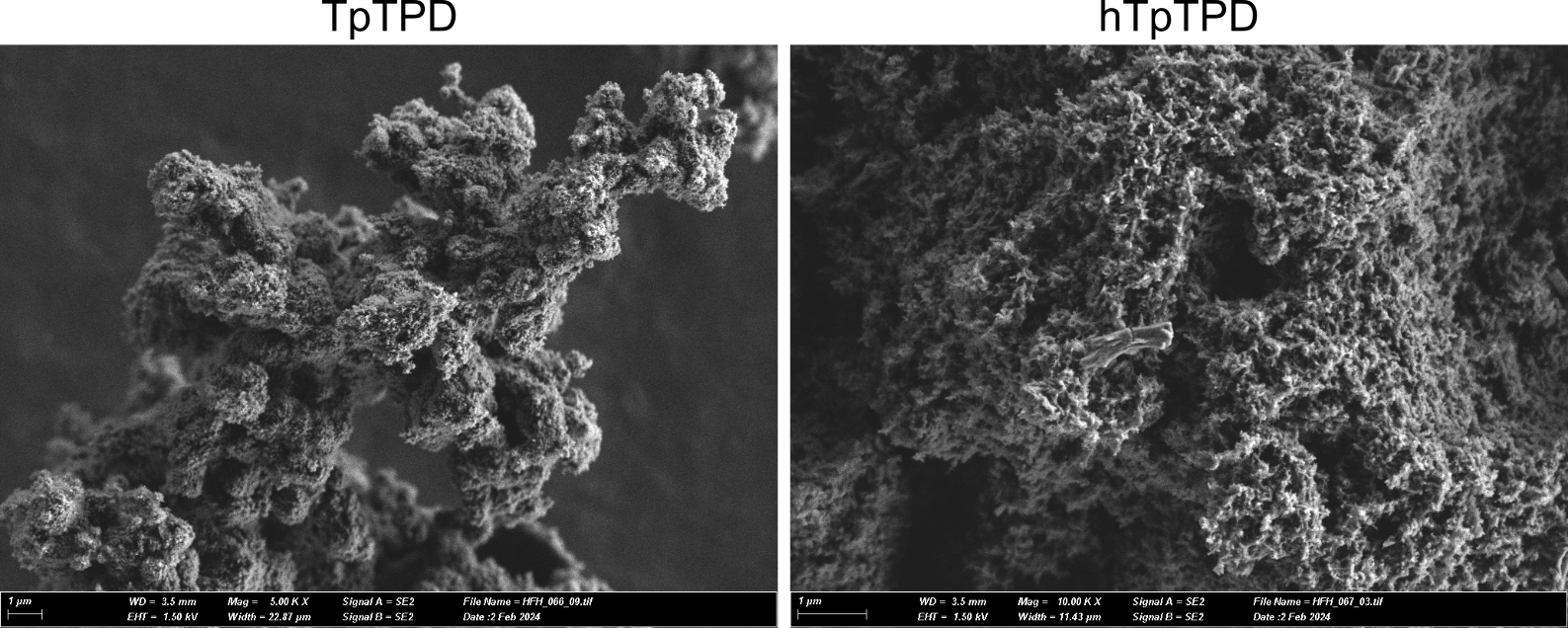


Figure S 41: SEM images of TpTPD and hTpTPD.


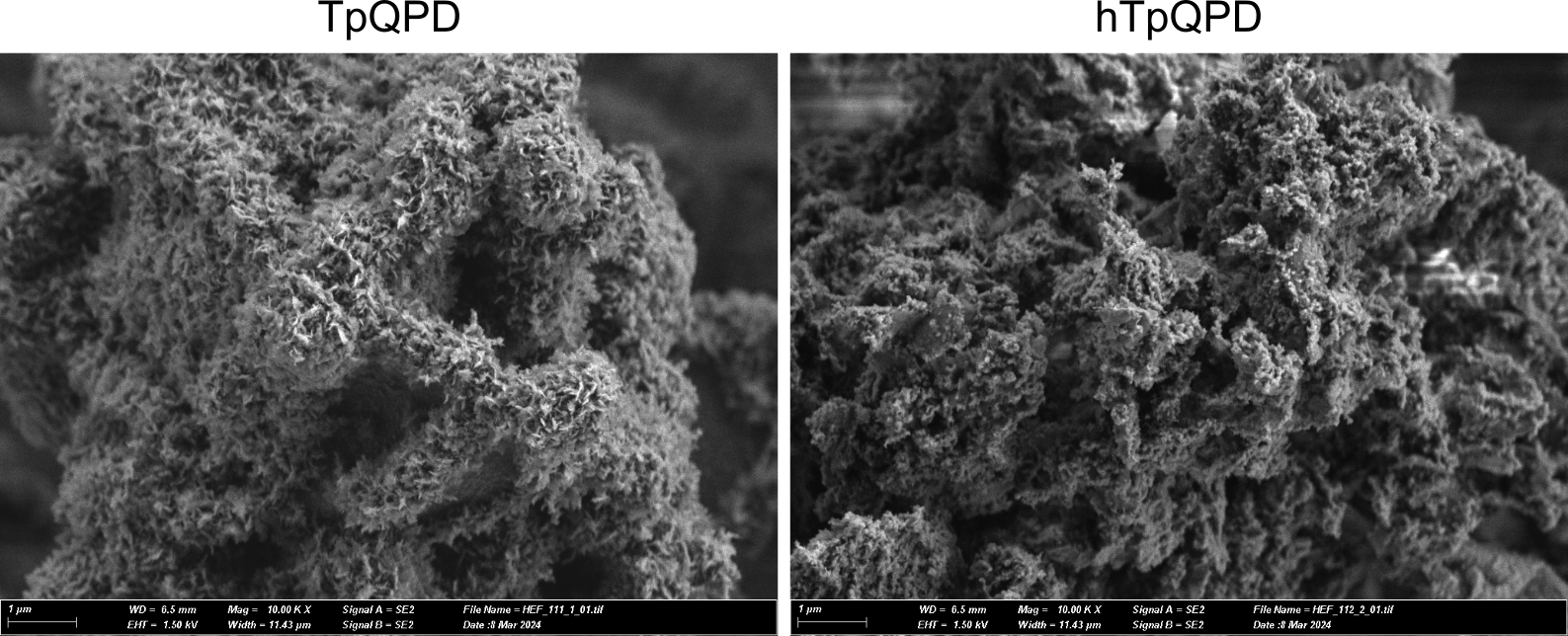


Figure S 42: SEM images of TpQPD and hTpQPD.


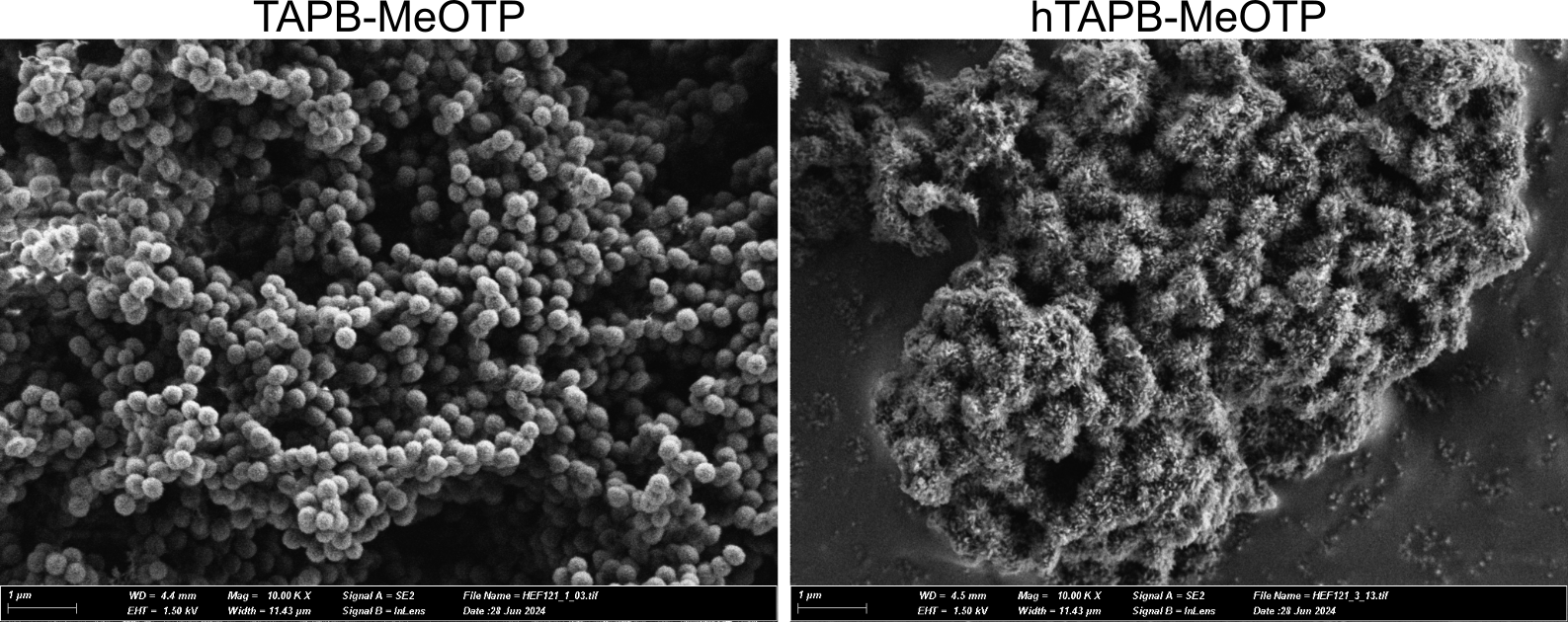


Figure S 43: SEM images of TAPB-MeOTP and hTAPB-MeOTP.


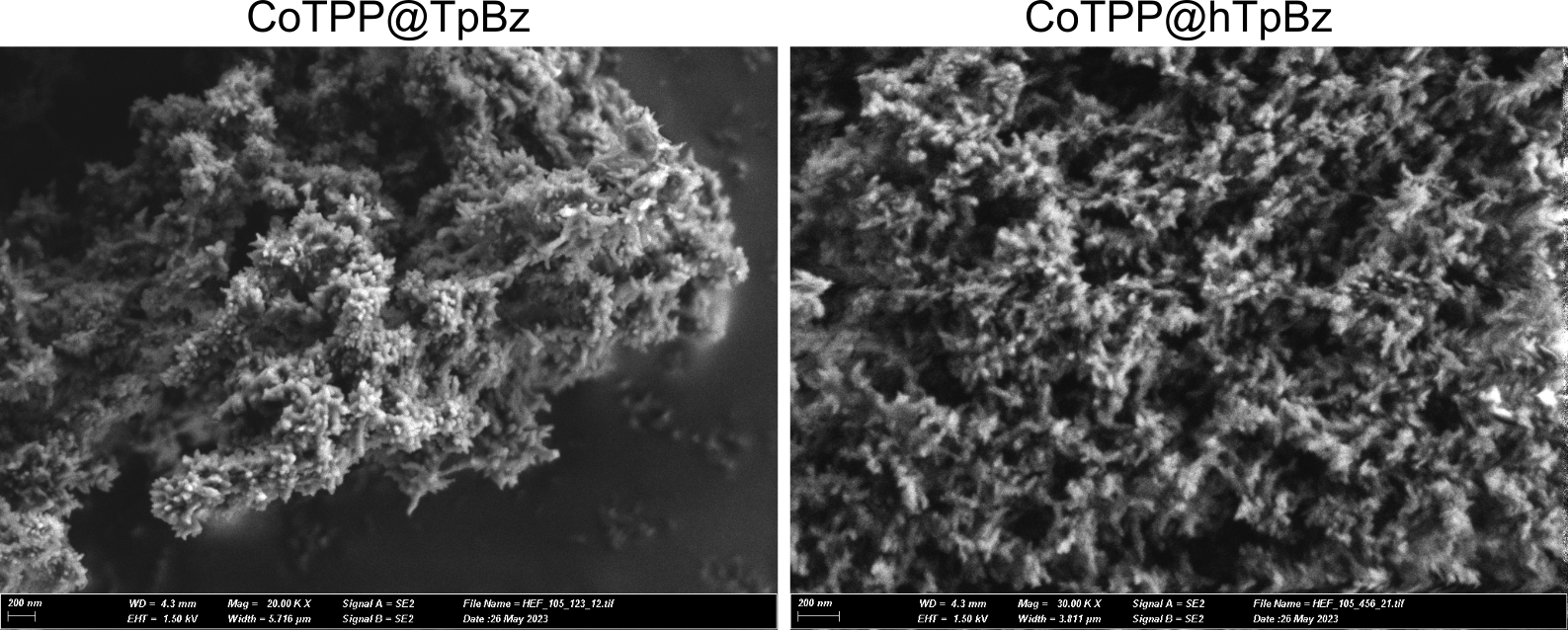


Figure S 44: SEM images of CoTPP loaded CoTPP@TpBz and CoTPP@hTpBz.


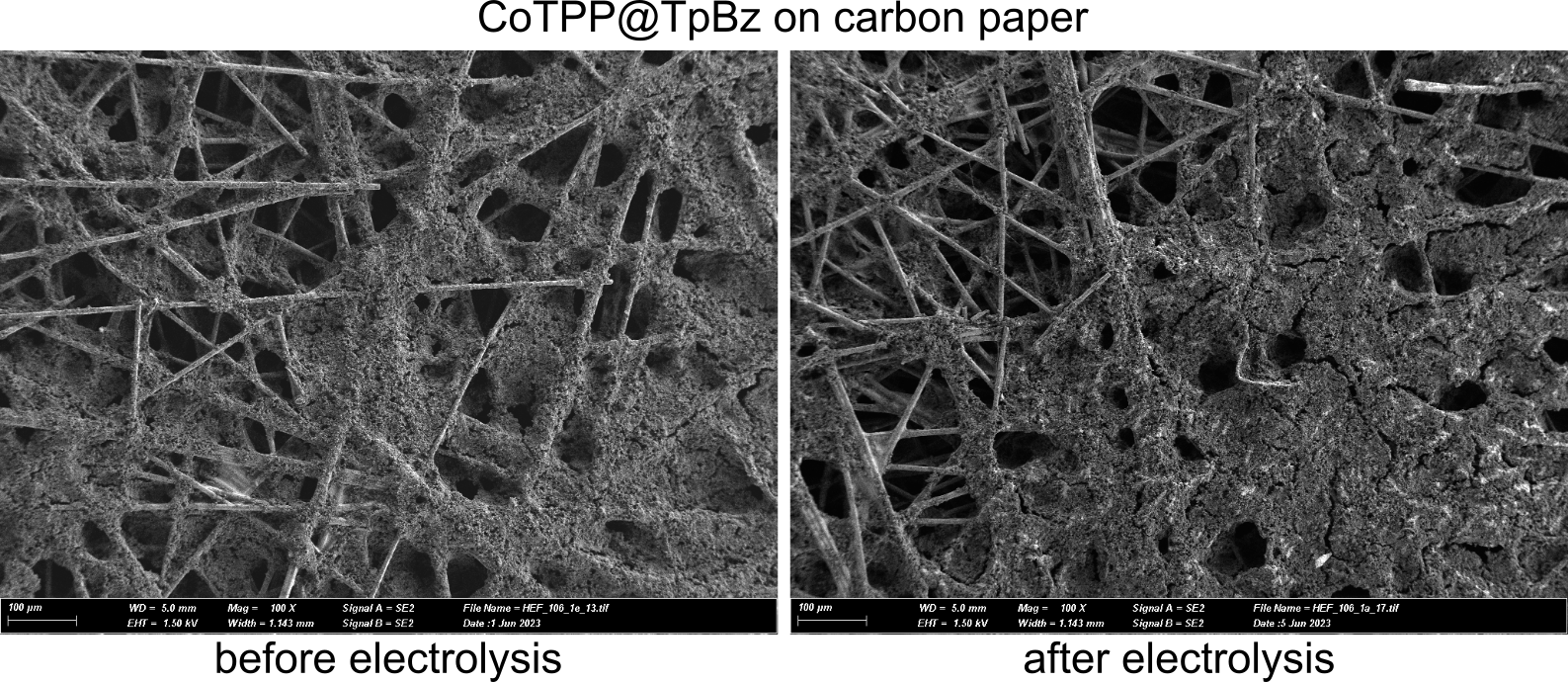


Figure S 45: SEM images of two electrodes loaded with CoTPP@TpBz ink before and after electrolysis. The electrochemical CO_2_ reduction was performed on the electrode on the right at −0.53 V and −0.93 V vs. RHE. Both images display similarities in morphology.


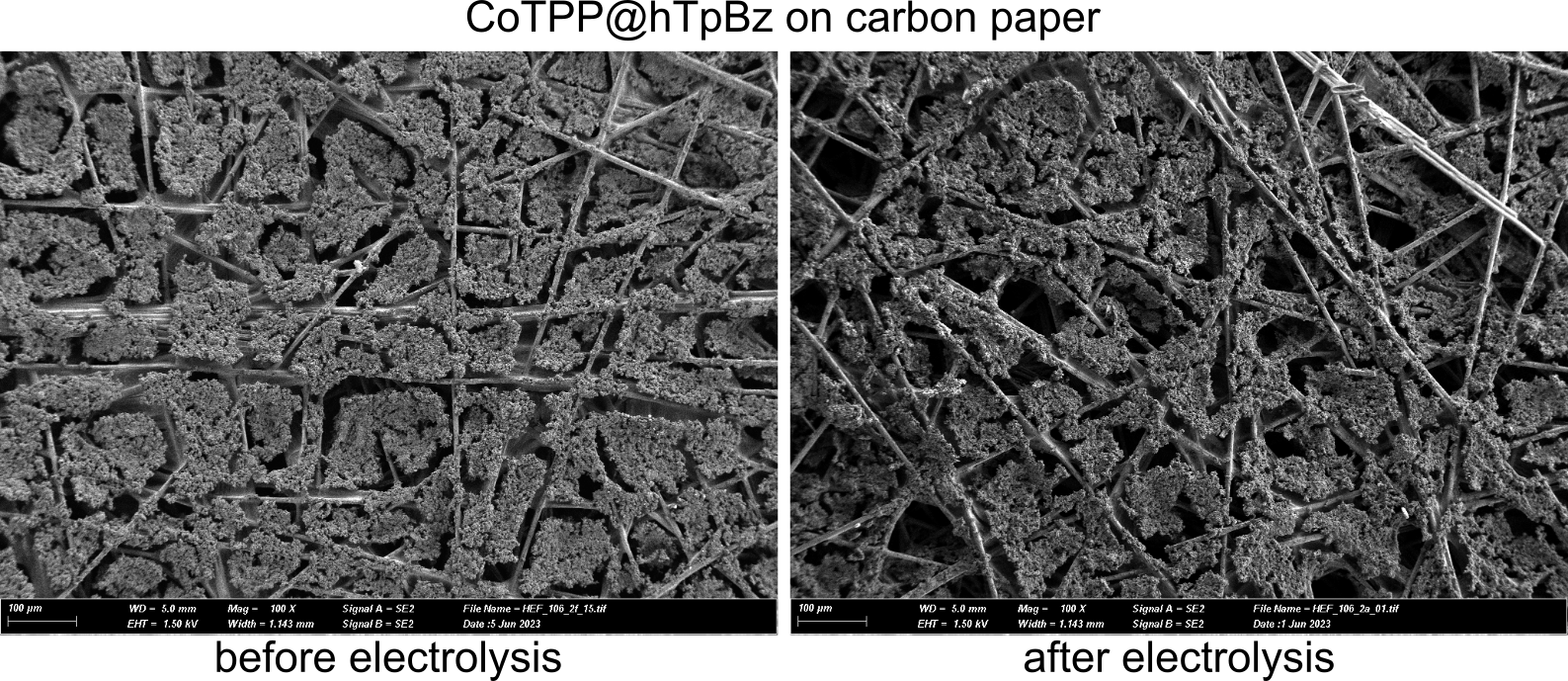


Figure S 46: SEM images of two electrodes loaded with CoTPP@hTpBz ink before and after electrolysis. The electrochemical CO_2_ reduction was performed on the electrode on the right at −0.53 V and −0.93 V vs. RHE. Both images display similarities in morphology.

**Dynamic Light Scattering (DLS)**

Figure S 47: Dynamic light scattering (DLS) of ZnO nanoparticles at different reaction conditions. In blue, the pristine ZnO NP suspension. In red, reaction conditions for the new synthetic approach in DMF at 90 °C for 24 h. In yellow, reaction conditions of a reported route via Sc(OTf)_3_ in acetonitrile (ACN).

**Electrochemical Data**

Figure S 48: Overview of cyclic voltamogramms of the (a) TpBz and (b) hTpBz *vs*. their CoTPP loaded counterparts in reference to a carbon paper electrode dropcasted with bare carbon black normalized by electrode area. Dashed lines indicating corresponding onset potentials. H-cell set-up with a scan rate of 20 mV s^−1^ in 0.5 M KHCO_3_, a Pt counter electrode and an Ag/AgCl reference electrode.

Figure S 49: Overview of the chronoamperometric measurements of CoTPP@TpBz along five different potentials normalized by electrode area.

Figure S 50: Overview of the chronoamperometric measurements of CoTPP@hTpBz along five different potentials normalized by electrode area.

Figure S 51: Overview of the chronoamperometric measurements of the bare control samples TpBz, ZnO@TpBz, hTpBz referenced to carbon paper at ‑0.73 V vs. RHE and normalized by electrode area. The carbon paper reference was prepared with a Nafion 117 and carbon black ink according to the aforementioned procedure without catalyst.

Figure S 52: Faradaic efficiencies of the bare COF control samples without CoTPP loading of TpBz, ZnO@TpBz, hTpBz referenced to carbon paper at ‑0.73 V *vs.* RHE and normalized by electrode area. The carbon paper reference was prepared with a Nafion 117 and carbon black ink according to the aforementioned procedure without catalyst.

Figure S 53: Initial partial current densities of both candidates at the first injection of the online GC analysis for the tested potentials in chronoamperometric measurements.

Figure S 54: Calculated turn over frequency (TOF) at initial current densities along five different potentials *vs.* RHE based on the assumption that all cobalt sites on the carbon paper electrode are catalytically active. Co amount was derived from the results of ICP-OES and the amount of ink dropcasted on the electrode.

Figure S 55: Overview of the Faradaic efficiencies of the CoTPP loaded (h)TAPB-MeOTP system. CoTPP loaded hTAPB-MeOTP exhibits a higher CO Faradaic efficiency at −0.93 V *vs.* RHE, aligning with the trend observed for CoTPP loaded (h)TpBz COFs.

Figure S 56: Overview of the Co amounts analyzed *via* ICP-OES of the CoTPP loaded (h)TAPB-MeOTP system. The rather large difference in Co amount between the two systems, which directly correlates with the catalyst loading, most likely also impacts the less pronounced performance boost in eCO_2_RR in contrast to the CoTPP@(h)TpBz system.

**References**

[1] L. Grunenberg, C. Keßler, T. W. Teh, R. Schuldt, F. Heck, J. Kästner, J. Groß, N. Hansen, B. V. Lotsch, *ACS Nano* **2024**, 18, 16091.

[2] J. Kärger, M. Avramovska, D. Freude, J. Haase, S. Hwang, R. Valiullin, *Adsorption* **2021**, 27, 453.

[3] A. Coelho, *J. Appl. Crystallogr.* **2018**, 51, 210.

[4] G. S. Pawley, *J. Appl. Crystallogr.* **1981**, 14, 357.

[5] R. W. Cheary, A. Coelho, *J. Appl. Crystallogr.* **1992**, 25, 109.

[6] S. T. Emmerling, R. Schuldt, S. Bette, L. Yao, R. E. Dinnebier, J. Kästner, B. V. Lotsch, *J. Am. Chem. Soc.* **2021**, 143, 15711.

[7] T. C. Huang, H. Toraya, T. N. Blanton, Y. Wu, *J. Appl. Crystallogr.* **1993**, 26, 180.

[8] J. W. M. Osterrieth, J. Rampersad, D. Madden, N. Rampal, L. Skoric, B. Connolly, M. D. Allendorf, V. Stavila, J. L. Snider, R. Ameloot, J. Marreiros, C. Ania, D. Azevedo, E. Vilarrasa-Garcia, B. F. Santos, X.-H. Bu, Z. Chang, H. Bunzen, N. R. Champness, S. L. Griffin, B. Chen, R.-B. Lin, B. Coasne, S. Cohen, J. C. Moreton, Y. J. Colón, L. Chen, R. Clowes, F.-X. Coudert, Y. Cui, B. Hou, D. M. D'Alessandro, P. W. Doheny, M. Dincă, C. Sun, C. Doonan, M. T. Huxley, J. D. Evans, P. Falcaro, R. Ricco, O. Farha, K. B. Idrees, T. Islamoglu, P. Feng, H. Yang, R. S. Forgan, D. Bara, S. Furukawa, E. Sanchez, J. Gascon, S. Telalović, S. K. Ghosh, S. Mukherjee, M. R. Hill, M. M. Sadiq, P. Horcajada, P. Salcedo-Abraira, K. Kaneko, R. Kukobat, J. Kenvin, S. Keskin, S. Kitagawa, K.-i. Otake, R. P. Lively, S. J. A. DeWitt, P. Llewellyn, B. V. Lotsch, S. T. Emmerling, A. M. Pütz, C. Martí-Gastaldo, N. M. Padial, J. García-Martínez, N. Linares, D. Maspoch, J. A. Suárez del Pino, P. Moghadam, R. Oktavian, R. E. Morris, P. S. Wheatley, J. Navarro, C. Petit, D. Danaci, M. J. Rosseinsky, A. P. Katsoulidis, M. Schröder, X. Han, S. Yang, C. Serre, G. Mouchaham, D. S. Sholl, R. Thyagarajan, D. Siderius, R. Q. Snurr, R. B. Goncalves, S. Telfer, S. J. Lee, V. P. Ting, J. L. Rowlandson, T. Uemura, T. Iiyuka, M. A. van der Veen, D. Rega, V. Van Speybroeck, S. M. J. Rogge, A. Lamaire, K. S. Walton, L. W. Bingel, S. Wuttke, J. Andreo, O. Yaghi, B. Zhang, C. T. Yavuz, T. S. Nguyen, F. Zamora, C. Montoro, H. Zhou, A. Kirchon, D. Fairen-Jimenez, *Adv. Mater.* **2022**, 34, 2201502.

[9] K. Endo, A. Raza, L. Yao, S. Van Gele, A. Rodríguez-Camargo, H. A. Vignolo-González, L. Grunenberg, B. V. Lotsch, *Adv. Mater.* **2024**, n/a, 2313197.

[10] X. Zhao, P. Pachfule, S. Li, T. Langenhahn, M. Ye, C. Schlesiger, S. Praetz, J. Schmidt, A. Thomas, *J. Am. Chem. Soc.* **2019**, 141, 6623.

[11] J. H. Chong, M. Sauer, B. O. Patrick, M. J. MacLachlan, *Org. Lett.* **2003**, 5, 3823.

[12] L. Yao, A. Rodríguez-Camargo, M. Xia, D. Mücke, R. Guntermann, Y. Liu, L. Grunenberg, A. Jiménez-Solano, S. T. Emmerling, V. Duppel, K. Sivula, T. Bein, H. Qi, U. Kaiser, M. Grätzel, B. V. Lotsch, *J. Am. Chem. Soc.* **2022**, 144, 10291.

[13] L. Grunenberg, C. Keßler, T. W. Teh, R. Schuldt, F. Heck, J. Kästner, J. Groß, N. Hansen, B. V. Lotsch, doi: 10.26434/chemrxiv-2024-d9rmq This content is a preprint and has not been peer-reviewed.
